# Common biochemical properties of metabolic genes recurrently dysregulated in tumors

**DOI:** 10.1186/s40170-020-0211-1

**Published:** 2020-05-08

**Authors:** Krishnadev Oruganty, Scott Edward Campit, Sainath Mamde, Costas A. Lyssiotis, Sriram Chandrasekaran

**Affiliations:** 1 Department of Biomedical Engineering, University of Michigan, Ann Arbor, MI 48105 USA; 2 Present Address: Genpact, New York, NY 10036 USA; 3 Program in Chemical Biology, University of Michigan, Ann Arbor, MI 48105 USA; 4 Department of Molecular and Integrative Physiology, University of Michigan Medical School, Ann Arbor, MI 48109 USA; 5 Department of Internal Medicine, Division of Gastroenterology and Hepatology, University of Michigan Medical School, Ann Arbor, MI 48109 USA; 6 Rogel Cancer Center, University of Michigan Medical School, Ann Arbor, MI 48109 USA

**Keywords:** Metabolic modeling, Genomics, Transcriptomics, Machine learning

## Abstract

**Background:**

Tumor initiation and progression are associated with numerous metabolic alterations. However, the biochemical drivers and constraints that contribute to metabolic gene dysregulation are unclear.

**Methods:**

Here, we present MetOncoFit, a computational model that integrates 142 metabolic features that can impact tumor fitness, including enzyme catalytic activity, pathway association, network topology, and reaction flux. MetOncoFit uses genome-scale metabolic modeling and machine-learning to quantify the relative importance of various metabolic features in predicting cancer metabolic gene expression, copy number variation, and survival data.

**Results:**

Using MetOncoFit, we performed a meta-analysis of 9 cancer types and over 4500 samples from TCGA, Prognoscan, and COSMIC tumor databases. MetOncoFit accurately predicted enzyme differential expression and its impact on patient survival using the 142 attributes of metabolic enzymes. Our analysis revealed that enzymes with high catalytic activity were frequently upregulated in many tumors and associated with poor survival. Topological analysis also identified specific metabolites that were hot spots of dysregulation.

**Conclusions:**

MetOncoFit integrates a broad range of datasets to understand how biochemical and topological features influence metabolic gene dysregulation across various cancer types. MetOncoFit was able to achieve significantly higher accuracy in predicting differential expression, copy number variation, and patient survival than traditional modeling approaches. Overall, MetOncoFit illuminates how enzyme activity and metabolic network architecture influences tumorigenesis.

## Background

Tumors reprogram cellular metabolism to support uncontrolled cell proliferation [1–4]. A common metabolic reprogramming exhibited by tumors is the Warburg effect, where malignant cells shift metabolic flux away from oxidative phosphorylation to glycolysis [5, 6]. Although diverse tumors exhibit common metabolic features such as the Warburg effect, recent meta-analysis studies of cancer transcriptome and metabolome revealed that metabolic changes are highly heterogeneous across different tumor types [7]. This suggests that tumors have diverse metabolic objectives. Given the enormous redundancy in the metabolic network [8, 9], we hypothesized that cancer cells make systemic changes at several regulatory layers, resulting in few key changes in the metabolic network. We hence examined frequently dysregulated metabolic genes using a multi-scale systems-biology approach to determine if there are common features that contribute to metabolic dysregulation in tumors.

MetOncoFit is a data-driven approach we developed to identify the topological and biochemical features that are predictive of metabolic alterations in tumors. While recent meta-analyses have interpreted tumor-omics data using known metabolic pathways and metabolic network models [7, 10–13], these studies do not provide insights on how enzyme kinetic properties or network topology can impact metabolic reprogramming. Our approach goes beyond existing methods by focusing on an extensive set of biochemical, topological, and metabolic factors that are analyzed together for the first time. The MetOncoFit approach accounts for a broad range of attributes including enzyme catalytic activity, expression levels, metabolic pathway membership, topological connectivity to biomass and media components, and metabolic flux from in silico knockout experiments.

Through meta-analysis of copy number variation (CNV) and transcriptomic data from various cancer databases using MetOncoFit, our study demonstrates how specific biochemical features such as catalytic activity are predictive of metabolic gene dysregulation across various cancer types. This finding explains why some genes within the same pathway show vast differences in the frequency of dysregulation. Further, the inclusion of novel topological and biochemical features enabled our approach to achieve significantly higher accuracy in predicting dysregulated genes compared to traditional grouping of genes into pathways. MetOncoFit revealed common pan-cancer objectives of metabolic dysregulation and accurately predicted how dysregulation of metabolic gene activity will alter tumor fitness and patient survival.

For any new transcriptomics or CNV dataset, MetOncoFit can be deployed to uncover the relative importance of various metabolic and topological features in predicting dysregulation observed in the dataset. Quantifying the relative importance of various factors can potentially be significant for developing metabolic therapeutics. For example, if the network topology factors, such as connectivity to key nutrients, dominate in a given tumor, then focusing on eliminating specific nutrients can be an effective strategy. In contrast, if dysregulated enzyme activity best explains the metabolic phenotype, then treatments should focus on reducing the activity of a specific enzyme or pathway. This approach will ultimately improve our ability to predict targeted metabolic therapies.

## Results

### Biochemical and network features used in MetOncoFit

**Fig. 1:**
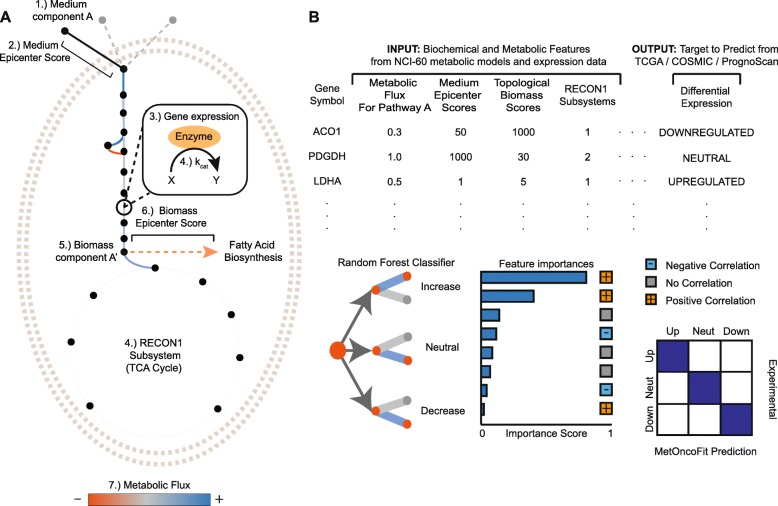
Overview of the MetOncoFit Approach. **a** The MetOncoFit model consists of 142 metabolic features (Additional file 1: Table S1). These features include biochemical properties (e.g., catalytic activity (*k*_cat_)), topological parameters from the RECON1 network model (e.g., biomass epicenter score), and dynamic properties computed from the NCI-60 cancer cell line metabolic models (e.g., reaction flux). These features for each gene are used to make predictions on its impact on tumor fitness in a specific cancer context. **b** The sample dataset in the figure shows the input matrix for MetOncoFit. The columns span the 142 features used in our model. The features are inputted into a random forest classification algorithm and it outputs ternary predictions (increased/neutral/decreased) for copy number variation, differential gene expression, and cancer patient survival based on the predicted impact of gene activity on tumor fitness. We evaluated MetOncoFit performance using 10-fold cross-validation. MetOncoFit also ranks features based on their predictive importance and outputs the direction of impact with expression, survival, or CNV (i.e., positive or negative correlation)

MetOncoFit uses biochemical and network-level properties of a metabolic gene to predict if it will be dysregulated in tumors. Two objectives guided the choice of features and datasets used in our model. First, we identified metabolic features that could affect cancer cell fitness. Second, we shortlisted features that can be easily quantified and are widely available. We grouped the feature set for each gene into three major classes—biochemical, topological, and dynamic properties described below (Fig. 1). The topological and dynamic parameters quantify the position of each enzyme in the network and its impact on network fluxes, respectively, while the biochemical properties quantify the relative abundance and intrinsic activity of each enzyme. In total, 142 features were used as input for MetOncoFit. These features can help identify common properties of genes that are frequently dysregulated in tumors.

#### Topological features

We used pathway association and network-level characteristics as topological features for each gene in our model. Tumors frequently upregulate transporters to increase nutrient uptake [14, 15]. Yet traditional pathway annotations do not capture the network-level proximity of enzymes to nutrients and metabolic precursors. We hence derived this topological parameter using the human genome-scale metabolic reconstruction, RECON1, which contains 3747 reactions, 1496 open reading frames, 2004 proteins, and 2766 metabolites [16]. This model represents the mechanistic relationships between genes, proteins, and metabolites in a human cell. This network was used to calculate the shortest path from 33 exogenous media components, such as glucose and amino acids, to reaction products catalyzed by a metabolic enzyme. Similarly, we also calculated the total distance from the metabolic reaction to 44 individual biomass components (such as nucleotides and lipids) and the total distance from the reaction to all biomass and media components. We call these the topological biomass epicenter score and topological media epicenter score, respectively. Finally, we considered the canonical metabolic pathway association (e.g., glycolysis or citric acid cycle) and the metabolic subnetwork (central carbon metabolism, intermediate metabolism, and secondary metabolism) associations as topological features within our model.

#### Dynamic features

In addition to the static topological network attributes that are fixed for each gene in a condition, we analyzed the impact of each enzyme on the overall flux through each reaction in the network using flux balance analysis (FBA) [17]. FBA optimizes a cellular objective, usually the conversion of nutrients to biomass. FBA has been applied successfully to predict metabolic behaviors of various cancer cells and tissues [10, 18–20]. To identify metabolic reactions that are differentially active in specific cancer tissues, we used the NCI-60 cell-line metabolic models from Yizhak et al. [21]. For each gene in the cancer-specific metabolic model, we calculated the average metabolic flux from systematic single gene deletions in the model through 52 metabolic pathways (see the “Methods” section, Additional file 1: Table S1).

#### Biochemical activity features

We used the enzyme catalytic activity (*k*_cat_) and expression levels as the biochemical activity features for each gene in our model, which are equally important in determining reaction flux. Enzyme levels in a cell are fine-tuned to convert media components to biomass. In contrast to expression levels, the role of the catalytic activity on dysregulation frequency is not known. The catalytic activity values for each enzyme were taken from the manually curated SABIO Biochemical Reaction database [22]. Cell-type specific gene expression for each metabolic gene was obtained from the NCI-60 panel of cancer cell lines [23].

### MetOncoFit accurately predicts dysregulated metabolic genes using biochemical and network properties

The MetOncoFit approach operates on the hypothesis that fundamental biochemical and network level properties of a metabolic gene are predictive of dysregulations leading to increased fitness of tumors. We identified genes that impact tumor fitness as those that are recurrently differentially expressed in matched tumor-normal samples from TCGA, exhibit recurrent copy number changes in COSMIC database, or if their activity is significantly associated with cancer patient survival in PrognoScan database [24, 25]. Overall, our datasets included data for 904 metabolic genes from 4459 transcriptomics samples with at least 500 samples for each tumor type, CNV data from 4415 samples, and survival data based on 6185 samples (Additional file 1: Table S2).

MetOncoFit trains a machine learning algorithm, random forests, on the topological, dynamic, and biochemical activity features to predict three prognostic markers of a gene’s impact on tumor fitness: differential metabolic gene expression, copy number variation, and cancer patient survival (see the “Methods” section). We classified genes into those that had increased, decreased, or had no impact on tumor fitness for each of the three markers, based on their biochemical and network-level properties (Fig. 1). Due to the large availability of cancer cell line datasets, we wanted to see if our machine learning approach can predict the dysregulated genes in patients using in silico models generated from the NCI-60 cancer cell line panel.

We evaluated the performance of MetOncoFit using data from nine different cancers: breast, B-cell lymphoma, ovarian, glioma, melanoma, prostate, colon, non-small cell lung, and renal cancer. These nine cancers were chosen as they are represented in the NCI-60 cancer cell line panel, which has been extensively studied using transcriptomics, proteomics, and metabolomics. Curated genome-scale metabolic models for these cell lines are available [21].

We assessed the performance of MetOncoFit using 10-fold cross-validation, leave-one-cell line out, leave-one-feature-set out, and holdout validation for 30% of the dataset. We assessed accuracy using confusion matrices, precision-recall curves, and the area under the receiver operating characteristic (AUROC) curve for predicting each cancer prognostic target (Additional file 1: Tables S3–S5; see the “Methods” section). In holdout validation, 70% of the data was randomly selected to train the model while the remaining 30% was used to test the model accuracy for each iteration. MetOncoFit was able to achieve an accuracy of 90% for 10-fold cross accuracy while predicting differential expression, copy number variation, and patient survival. Predicting differential expression and patient survival across all cancers had an overall higher average accuracy (97% and 94%) across all cancers, while predicting CNV had a modest average accuracy of 79%. The holdout accuracies were similar (Additional file 1: Tables S3–S5), demonstrating that our predictions are robust. Overall, MetOncoFit is able to predict differential expression and patient survival with high sensitivity and is able to predict CNV with modest sensitivity. The results suggest that the features used for classification are generalizable and show consistently high performance in identifying the metabolic genes that are dysregulated in each of the nine cancer tissue models.

Next, we categorized the input feature dataset into three distinct sets, specifically the dynamic features, static topological features, and biochemical activity features, and held out each set to determine the impact of specific feature categories on MetOncoFit’s performance (Additional file 1: Table S6). While biochemical activity features strongly contributed to MetOncoFit performance as expected, static topological features contributed as much, if not more, than the biochemical activity features to MetOncoFit’s accuracy across all nine cancer tissues and the pan cancer model (Additional file 1: Table S6). This suggests that the metabolic gene position within the network is an important attribute that influences dysregulation during tumorigenesis.

In the subsequent sections, we discuss our results on three cancer types: breast cancer, non-small cell lung cancer (NSCLC), and melanoma. The feature importance and performance details for the other six cancers are provided in Additional file 2: Figures S1–S6. The top features for each cancer can be interactively explored using the supplementary website (https://metoncofit.med.umich.edu).

### Biochemical and topological predictors of in vivo differential expression are shared across several tumor types

**Fig. 2:**
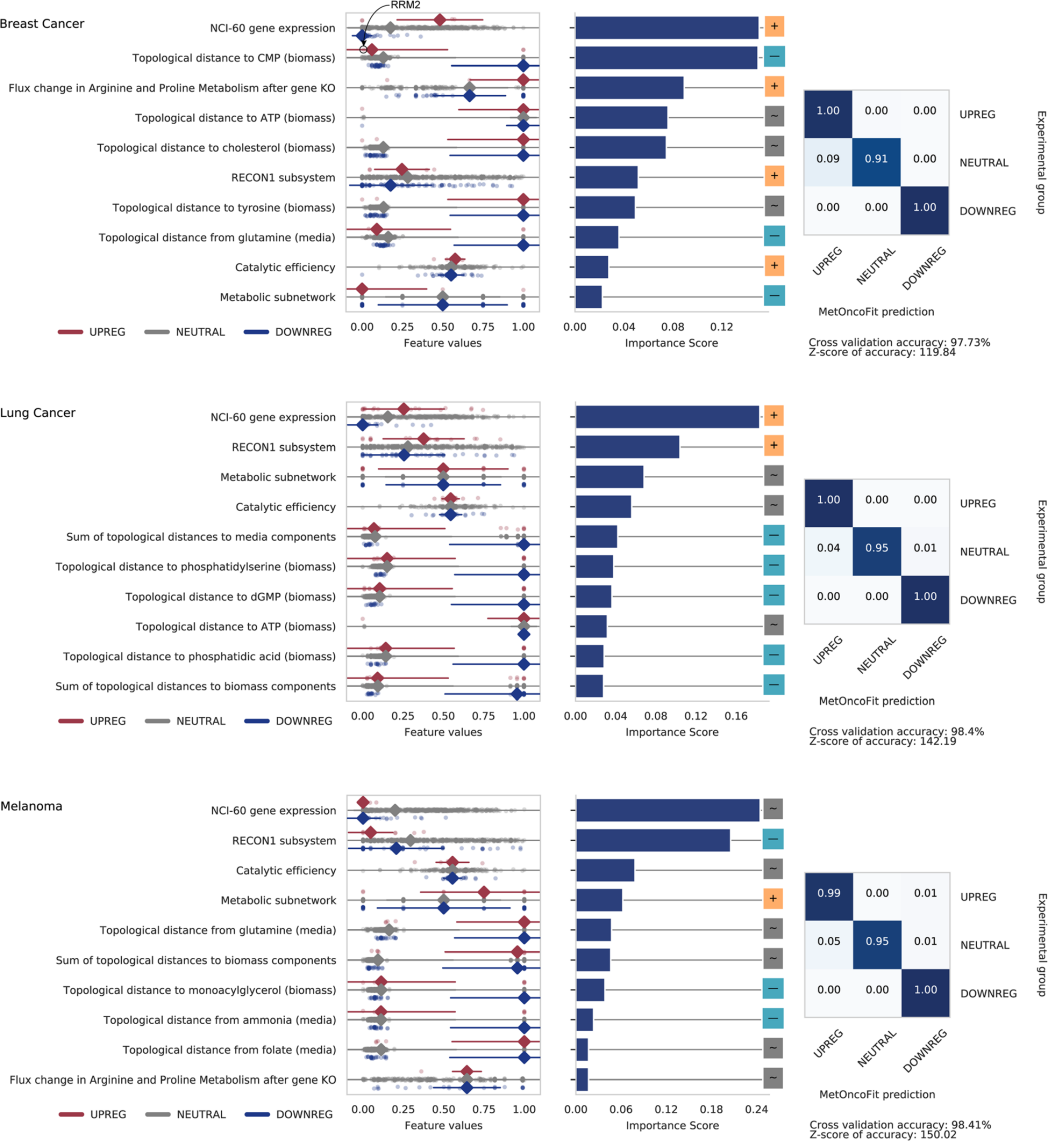
MetOncoFit accurately predicts differential expression in vivo using biochemical and topological properties of metabolic genes. The dot plots show the distribution of the values of each feature for the three classes of genes (upregulated, downregulated, or not differentially expressed in tumors compared to matched normal samples). The prominent diamond is the median value within the distribution, while the lines display the standard deviation from the median. Features are sorted based on their relative importance in predicting differential expression (top 10 shown). The [+], [~], and [−] square panels show the direction of the Pearson correlation value between differentially expressed classes and a given feature (see “Methods” section). Confusion matrices report MetOncoFit performance using 10-fold cross-validation; higher diagonal values indicate higher prediction accuracy of a specific class. Data for the three representative cancers were shown. See Additional file 2: Figures S3–S8 for corresponding data for all nine cancer types. The supplementary website provides data for each gene. Top panel: features predictive of differential expression in breast cancer include NCI-60 gene expression levels, catalytic activity, and flux after gene knockout through arginine and proline metabolism. The topological distances to the biomass component—CMP, are negatively correlated with breast cancer differential expression; enzymes topologically closer to CMP, such as RRM2, were more likely to be upregulated. Middle panel: a similar set of top features found in breast cancer were predictive of differential expression in NSCLC as well. In addition, enzymes topologically closer to the biomass components dGMP and phosphatidic acid were more likely to be upregulated. Bottom panel: in melanoma, the topological distance from ammonia was found to be a top predictor that is negatively correlated with differential expression

MetOncoFit showed very high accuracy in predicting matched tumor samples differential expression from the TCGA cancer patient gene expression data (CV accuracy = 98–99% for breast cancer, NSCLC, and melanoma) (Fig. 2). Several topological features dominated the top 10 most important predictors of differential expression in each cancer. Although some of the top 10 predictors show weak correlation (*R* < 0.6) with differential expression, the random forest algorithm can combine multiple weak predictors together to create an accurate model.

The topological distance to the nucleotides—CMP and ATP, appear as the top biological features in breast cancer (Fig. 2). MetOncoFit suggests that the metabolic enzymes closer to these nucleotides were more likely to be upregulated. MetOncoFit was hence able to correctly predict the upregulation of RRM2, a breast cancer biomarker that catalyzes the formation of deoxyribonucleotides [26, 27] (Additional file 2: Figure S7).

The presence of numerous topological features as top predictors across all cancers supports the idea that the metabolic network topology strongly influences metabolic dysregulation. Furthermore, for many cancer types, the topological biomass score was significantly correlated with gene expression. While enzymes that are near the network center would be expected to be dysregulated due to their interconnectedness with other pathways, our findings suggest that enzymes farther from the center of the network are more likely to be dysregulated in cancers, resulting in altered nutrient uptake and biomass synthesis.

Furthermore, enzyme catalytic activity was found to be a top predictor of differential expression across all cancer types. This suggests that enzyme biochemical properties can influence tumor metabolic rewiring strategies. We found that there was a positive correlation between the catalytic activity and differential expression in many cancers, including ovarian cancer and NSCLC. For example, enzymes in glycolysis and TCA cycle with low catalytic activity, such as HK3, FBP2, and GCK (median *k*_cat_ = 29, 16.7, and 40.1 s^−1^), are more likely to be downregulated in tumors (Fig. 2). In contrast, enzymes in these pathways with high catalytic activity are more likely to be upregulated, such as TPI1, LDHA, and ENO1 (median *k*_cat_ = 1.44 × 10^7^, 308, and 115.25 s^−1^; see the “Methods” section) (Fig. 2). TPI1, LDHA, and ENO1 were also found to be frequently upregulated across various tumor types in a prior meta-analysis study [11]. Overall, these static biochemical and topological trends were shared across the nine tumors.

In addition, there was a significant correlation across all cancers between the NCI-60 gene expression and cancer patient differential expression from the TCGA. Genes with high NCI-60 cell line expression were upregulated in tumors. NCI-60 gene expression feature as a top predictor suggests that metabolic gene expression profiles from the NCI-60 cell line panel can be predictive of in vivo expression changes in tumors.

### Enzyme catalytic activity and flux through amino acid metabolism are top predictors of copy number gain and loss

**Fig. 3:**
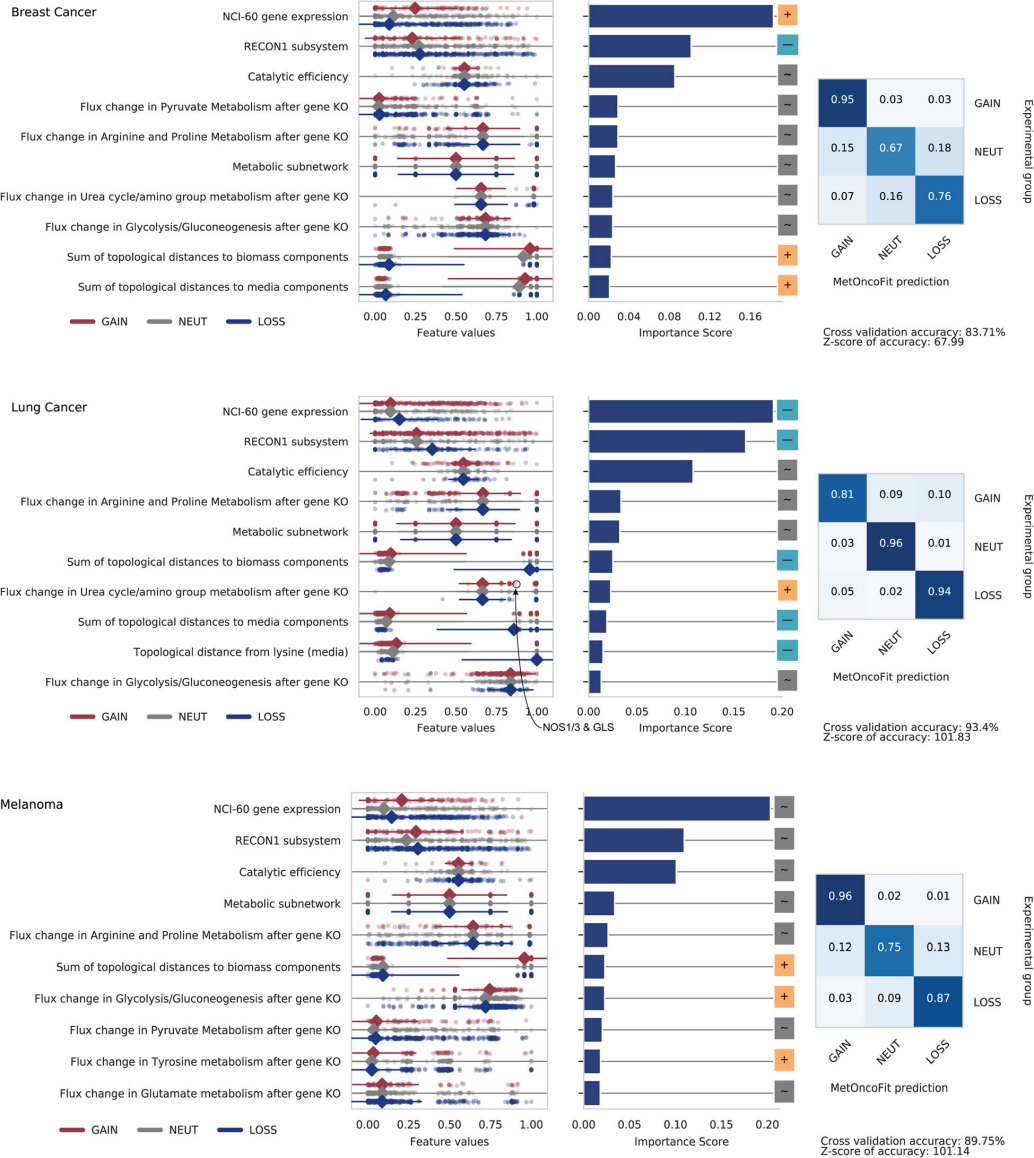
Predicting copy number variation using MetOncoFit. The dot plots show the distribution of the values of each feature for the three classes of genes (gain, loss, or neutral). Top 10 features predictive of copy number variation in breast cancer (top panel), NSCLC (middle panel), and melanoma (bottom panel) are shown. The 10-fold cross-validation accuracy is 85%, 94%, and 90% for breast cancer, NSCLC, and melanoma, respectively. Similar set of features were predictive of copy number variation in all three cancers including NCI-60 gene expression levels, catalytic activity, and flux after gene knockout through arginine and proline metabolism, and pyruvate metabolism. In NSCLC, increased flux through the urea cycle/amino group metabolism is associated with a gain in copy number. See Additional file 2: Figure S3 for corresponding data for all nine cancer types. The supplementary website provides data for each gene

While some metabolic enzyme copy number variants (CNV) such as PHGDH amplification have been associated with cancer [28], the link between CNV and its impact on cancer metabolism is still unclear. We included CNV as a target in our model to begin understanding how copy number gain or loss contributes to metabolic reprogramming in cancer cells. MetOncoFit showed high accuracy in predicting copy number gain/loss ratios (10-fold CV accuracy = 85%, 94%, 90% for breast cancer, NSCLC, and melanoma, respectively; Fig. 3). Similar to the MetOncoFit model for predicting differential expression, the topological media and biomass epicenters appear in the top 10 important features for predicting CNV in most cancers. MetOncoFit also identified the enzyme catalytic activity (*k*_cat_), the metabolic pathway association, the metabolic flux through arginine and proline metabolism, and flux through pyruvate metabolism as top 10 features contributing to CNV predictions for breast cancer, NSCLC, and melanoma.

Flux change in the urea cycle was positively correlated with the CNV ratio in NSCLC, suggesting a gain in copy number for those genes associated with those metabolic pathways (Fig. 3). The genes in these pathways—GLUL, GLS2, NOS1/3, GOT2, and ASL—displayed an overall gain in copy number in the COSMIC database, suggesting increased activity for these enzymes (Additional file 2: Figure S8). The copy number for these enzymes might be altered in lung cancer to support nitrogen metabolism. This metabolic rewiring strategy has been observed in KRAS/LBK1 mutant NSCLCs to manipulating nitrogen flow from ammonia to pyrimidine metabolism [29].

Flux through glycolysis plays an important role in melanoma initiation and maintenance [30, 31], and MetOncoFit was able to correctly predict that increased glycolytic flux is associated with copy number gain. MetOncoFit also predicted that copy number gains are associated with increased metabolic flux through tyrosine metabolism in melanoma. Previous studies have indicated that tyrosine and phenylalanine restriction in diet can suppress metastasis in in vitro and in vivo melanoma murine models [32, 33], suggesting that there is an increase in tyrosine metabolic activity for melanomas. Notably, MetOncoFit correctly predicted increased tyrosine metabolic flux, despite the CNV ratios for some genes in this pathway suggesting a loss in copy number. This suggests that MetOncoFit is able to infer rewiring due to enzymes upstream of a pathway rather than relying on genes in one pathway alone.

Gene expression fold change values in cancer patients show a positive correlation with CNVs in several studies [34, 35]. Hence, to further improve our accuracy, we retrained our model to include the TCGA cancer atlas gene expression fold change. MetOncoFit was able to predict CNV with higher accuracy after including TCGA expression data (10-fold CV accuracy = 92%, 98%, and 98%) for breast cancer, NSCLC, and melanoma, respectively (Additional file 2: Figure S9). We found that the top 10 important features in all three cancers essentially remained the same to the model without using TCGA expression. Additionally, the TCGA expression fold change was predicted to have the highest impact in the CNV prediction for all three cancers. In sum, these results suggest that MetOncoFit can accurately predict how metabolic gene copy number influences cancer metabolism.

### Enzyme catalytic activity and expression level are predictive of patient survival

**Fig. 4:**
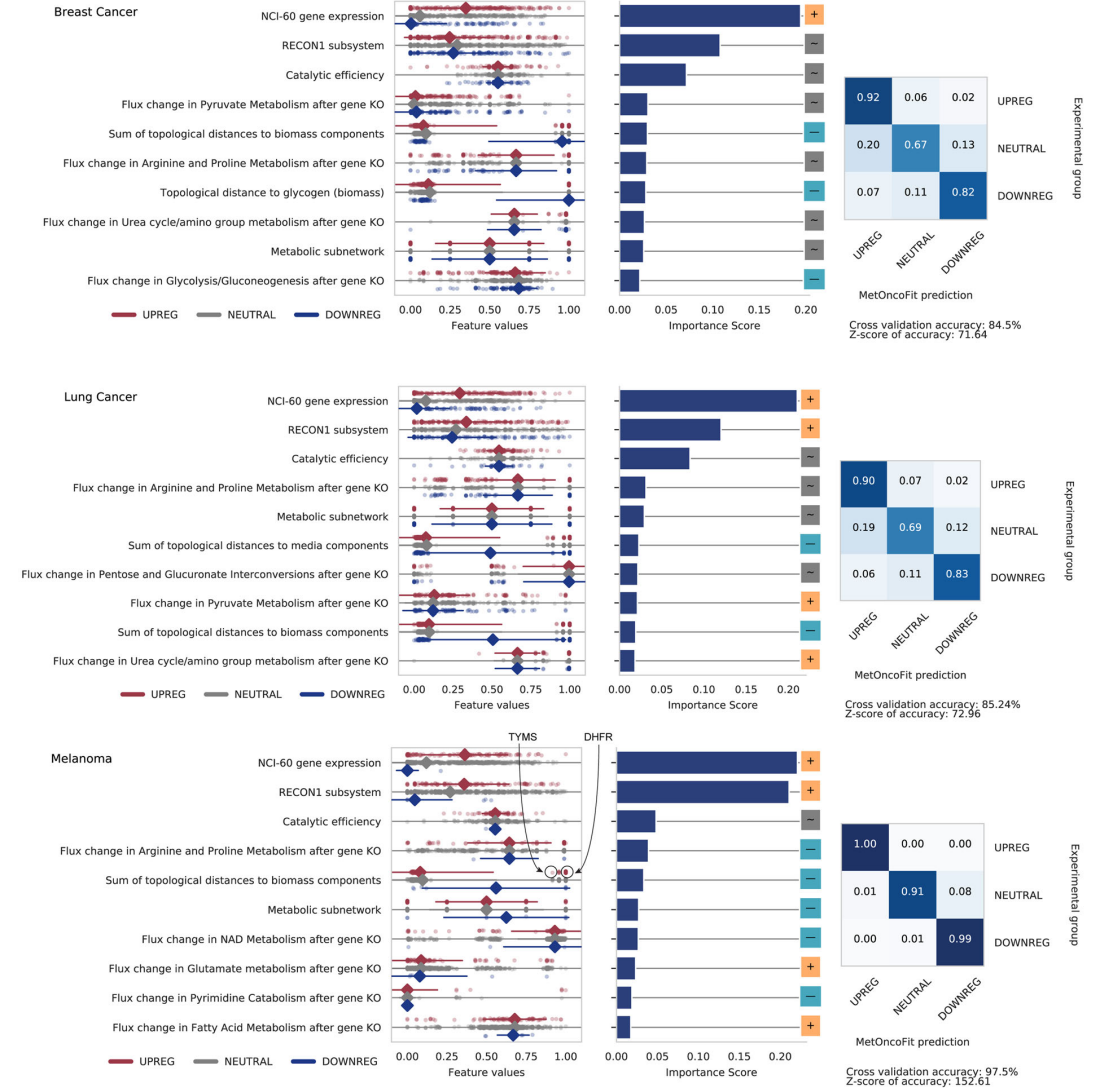
MetOncoFit identified topological and dynamic metabolic features that are predictive of gene’s activity on tumor fitness and patient survival. Genes were grouped into three classes based on their expression in poor survival group compared to good survival group—upregulated/neutral/downregulated (see the “Methods” section). The top 10 features predictive of a gene’s impact on patient survival in all three cancers include NCI-60 gene expression levels, catalytic activity, and flux through arginine and proline metabolism after gene knockout. The presence of catalytic activity and expression levels as top predictors suggest that enzyme activity is limiting tumor growth to a greater extent than specific metabolites. Genes that impact flux through arginine, proline, pyrimidine catabolism, and NAD metabolism when knocked out were found to be downregulated in melanoma patients with poor survival (bottom panel). In NSCLC (middle panel), genes that impact flux through pyruvate and the urea cycle/amino group metabolism were upregulated in patients with poor survival. The topological distance from glycogen biomass is negatively correlated with patient mortality in breast cancer (top panel), suggesting that upregulation of enzymes closer to these metabolites is associated with poor survival. The confusion matrices report the accuracy of model predictions using 10-fold cross validation. See Additional file 2: Figure S4 for corresponding data for all nine cancer types. The supplementary website provides data for each gene

MetOncoFit showed high accuracy for cancer patient survival prediction (10-fold CV accuracy = 86%, 85%, 98% for breast cancer, NSCLC, and melanoma, respectively) (Fig. 4). Metabolic features that improve cancer fitness are likely to have a detrimental effect on patient survival. The total biomass and media epicenter scores are consistently identified as the top 10 predictors of patient survival. This finding supports the assumption that cancer cells optimize biomass synthesis and nutrient uptake to increase their fitness. The total score of media and biomass components also show a bimodal distribution, suggesting that there are specific enzymes farther from the network center that are frequently dysregulated to enhance tumor fitness. For instance, DHFR, SQLE, and TYMS are located distant from the center of the network and are frequently upregulated in many tumors [36]. We further found that an increase in pyruvate metabolism flux has a positive impact on lung cancer patient survival (Fig. 4). Flux through pyruvate metabolism is a key metabolic branchpoint that controls the Warburg effect, which provides a metabolic benefit for cancer cell proliferation and serves as a prognostic marker in the clinic [5, 37]. Flux through pyruvate metabolism was upregulated in samples from lung cancer patients with increased survival. MetOncoFit found that increased glycolysis enhances tumor growth while increased pyruvate metabolism is associated with better patient survival. For example, LDHA is upregulated in lung cancers with poor survival and is associated with increased glycolytic flux [38].

Since gene expression and CNV in cancer patients are predictive of patient survival [39, 40], we also retrained MetOncoFit to predict survival using TCGA fold change data and copy number gain/loss ratios along with all 142 features. In addition to increasing the accuracy of our models, (Additional file 2: Figure S10; 10-fold CV accuracy = 93.7%, 97.9%, 99.3% for breast cancer, NSCLC, and melanoma, respectively), this analysis revealed specific metabolites that contribute to cancer patient survival. The model predicted that increased activity of enzymes topologically close to the nutrient glutamine is associated with increased melanoma patient mortality (Additional file 2: Figure S10). Glutamine is an essential metabolite for cancer cells involved in nitrogen and redox metabolism. Analysis of gene expression data also uncovered a strong association between ammonia metabolism and melanoma (Fig. 2), consistent with prior studies [41, 42]. MetOncoFit thus accurately recovers well-studied metabolic reprogramming associated with known oncogenic processes.

### Pan cancer model identifies pathways critical for all cancers

**Fig. 5:**
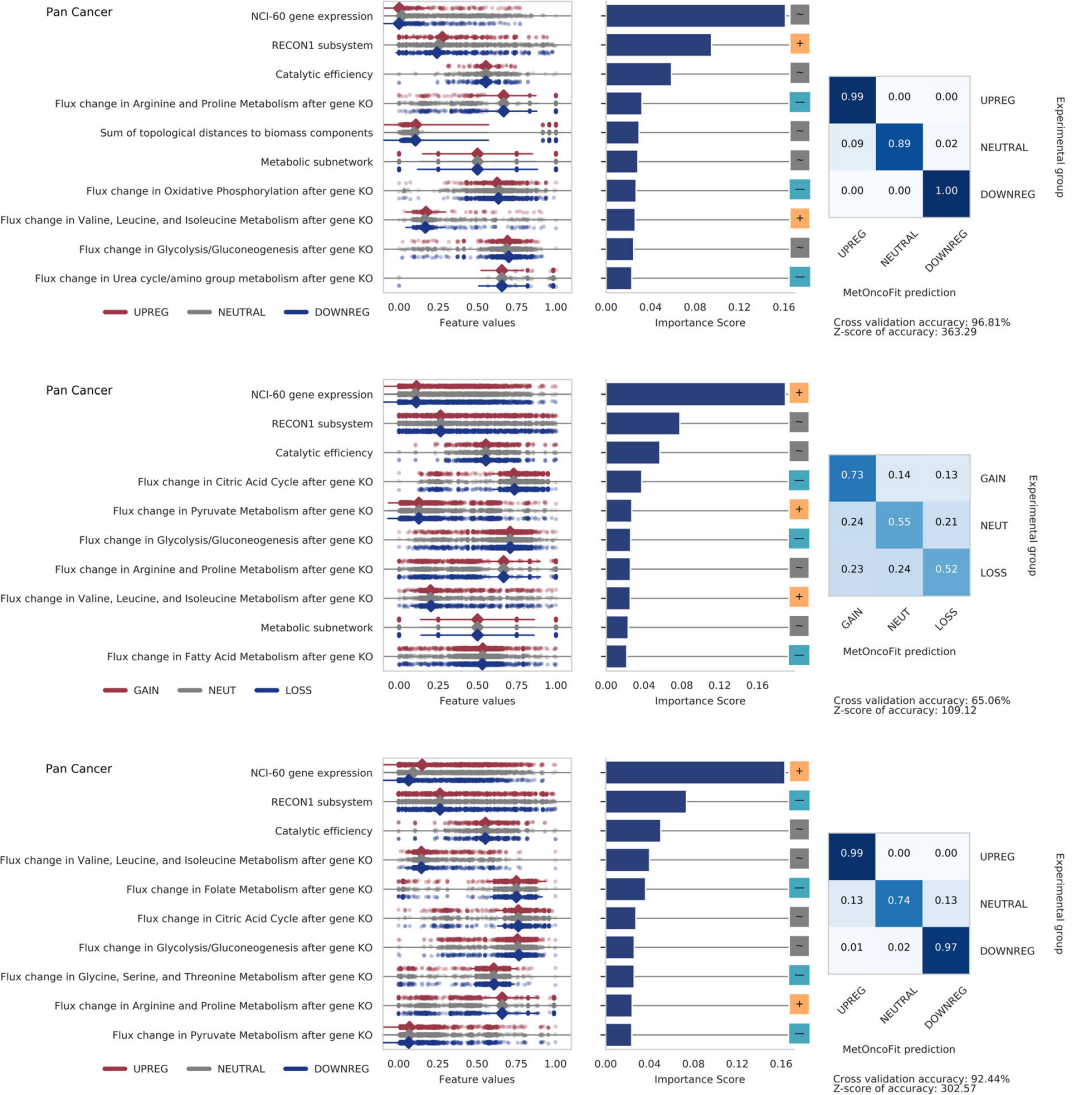
Pan cancer analysis identified common metabolic pathways contributing to tumor fitness across all cancer types. Top panel: predicting differential expression across all cancers. Genes that impact flux through oxidative phosphorylation are more likely to be downregulated in tumors. Middle panel: predicting copy number variation across all cancers. The very low predictive accuracy (65%) suggests that most features predictive of CNV are not conserved between cancers. Bottom panel: predicting patient survival across all cancers. Genes that impact flux through pyruvate, glycine, serine, and threonine metabolism, glycolysis, oxidative phosphorylation, arginine and proline metabolism, and folate metabolism are predictive of patient survival across all cancers. Notably, expression of genes highly expressed in NCI-60 cancer cell lines is associated with poor survival

To identify biological features that enhance fitness across all cancers, we trained a pan cancer model to predict the CNV gain/loss ratios, differential expression, and patient survival in all nine cancer types (Fig. 5). The performance of the pan cancer model was high for all fitness markers except for predicting CNV in comparison with cancer-specific models (10-fold CV accuracy = 97%, 64%, 93% for differential expression, CNV ratio, and survival, respectively). This suggests that the impact of copy number variation cannot be generalized and is tumor-specific. The top 10 features include NCI-60 cell line gene expression levels, catalytic activity, and impact on flux through glycolysis and amino acid synthesis pathways. Our pan cancer model also identified that dysregulation of central carbon metabolism and folate metabolism are important metabolic features that are conserved across all cancers. Both metabolic pathways undergo significant metabolic rewiring during cancer progression to support biomass, bioenergetics, and redox demands [36, 43]. Flux through folate metabolism generally contributes to decreased patient survival in our pan cancer model. Folate is used as a cofactor in purine synthesis [44] enabling cancer cells to keep up with cellular proliferation demands. Folate also plays a key role in changing the methylation patterns in DNA and histone proteins, altering gene expression to favor cancer cell survival [45]. Inhibiting folate metabolism was recently found to reduce proliferation of 16 different cancer cell types [11].

## Discussion

While recent genomic studies have cataloged several mutated genes and dysregulated enzymes, it is unclear why specific metabolic genes are recurrently dysregulated over others. Two prevailing hypotheses suggest that these recurrently dysregulated metabolic genes occupy key position in the network (the network hypothesis) or perform unique biochemical activities favorable for tumor fitness [1, 46] (enzyme activity hypothesis). While these assumptions are widely used in literature, a systematic analysis of the common attributes of recurrently mutated genes is lacking. To test these two hypotheses, we developed a data-driven framework called MetOncoFit to identify the common biochemical and network-level features of metabolic genes that impact tumor fitness. Our analysis using MetOncoFit supports both these assumptions, although the relative importance changes with tumor type. We identified tumors that are limited primarily by either substrates or enzyme activity, which can lead to distinct treatment options such as nutrient depletion or enzyme inhibition, respectively.

We first validated the MetOncoFit approach by assessing its accuracy in predicting differential expression and CNV in tumors from in vivo samples*.* Our analysis across nine different cancers revealed that the biochemical, topological, and flux features were sufficient to predict expression and CNV features with accuracies close to 90% and 80%, respectively, across all nine tumor types (Additional file 1: Table S3). Similarly, by identifying metabolic alterations that favor increased or decreased proliferation, we predicted with 90% accuracy the impact of these metabolic alterations on cancer patient survival (Additional file 1: Table S5). Overall, MetOncoFit accurately quantifies the impact of enzyme activity and metabolic network attributes on tumor fitness.

Our analysis revealed three key insights on cancer metabolism and tumor evolution. First, topological features were highly predictive of dysregulation. MetOncoFit revealed that enzymes away from the center of the network towards biomass components were more likely to be upregulated in tumors. Membrane transporters control substrate availability and are frequently overexpressed in cancer cells [3]. Our topological network analysis also identified limiting nutrients and biomass components. MetOncoFit predicted that enzymes topologically close to the synthesis of nucleotides such as cytosine monophosphate (CMP) were more likely to be upregulated in breast cancer and also appear as a top 10 feature in melanoma (Fig. 2). Thymidylate synthase (TYMS) is a classic example of an important metabolic enzyme that is recurrently dysregulated in several tumors to support DNA synthesis and affects patient survival [18]. Similarly, our model suggests that breast cancers may also upregulate enzymes that produce other pyrimidines, such as cytosine. This suggests that cancers upregulate enzymes that are bottleneck biomass synthesis. A similar observation on the impact of network location was made based on a meta-analysis of mutations across human diseases [46]. While this meta-analysis study focused on overall network topology alone across numerous diseases, our study quantifies the relative predictive power of specific topological factors in relation to other biochemical and network factors for individual tumor types.

Second, the enzyme catalytic activity (*k*_cat_) is predictive of change in enzyme expression levels in tumors, suggesting that intrinsic enzyme properties can influence metabolic rewiring strategies. The enzyme catalytic activity and enzyme expression are directly proportional to metabolic flux, and therefore altering the expression levels of enzymes with high enzyme catalytic activity results in flux rewiring with reduced protein cost. While the impact of aberrant expression of metabolic enzymes leading to oncogenic rewiring is widely documented, the role of the catalytic activity on dysregulation frequency is not known. We found that enzymes with high catalytic activity were more likely to show increased activity across most cancers through copy number gain or increased gene expression.

Finally, heterogeneous changes in multiple enzymes in tumors resulted in few key changes at the overall network level. This overarching framework of increased tumor fitness helps unify the highly diverse alterations observed in tumors. Several top predictive features for predicting CNV were also predictive of differential expression, suggesting that diverse mechanisms are used to achieve the same fitness goal. While the tumor fitness optimization assumption is a promising approach for understanding cancer metabolism, looking at individual-omics datasets alone may not reveal the optimal network state, as multiple complementary mechanisms are used to achieve optimal fitness.

While MetOncoFit was able to reveal key insights into several cancer metabolic rewiring strategies, there are limitations to the interpretability of our data-driven approach, as there are with all models. First, while differential expression and patient survival models had high accuracy, copy number variation could not be predicted with high accuracy in the pan cancer model, suggesting that CNVs cannot be generalized across cancers and a more stratified approach could be better at explaining CNVs. Further, some metabolic rewiring strategies are likely to be patient-specific and personalized cancer metabolic models can enable single patient analyses in the future [47, 48]. MetOncoFit identified enzyme catalytic activity to be a top predictor despite the lack of data (missing values) for several enzymes, suggesting that new technologies that would allow us to estimate these enzymatic parameters in a high-throughput manner would further increase model accuracy. Finally, metabolism is a dynamic process occurring over a wide timescale and is controlled by several regulatory levels. Incorporating additional parameters into metabolic modeling, such as post-translational modifications, proteomics, metabolomics, epigenetic markers, and metabolite feedback would further improve our ability to understand these diverse rewiring strategies in cancer cells [18, 48, 49].

## Conclusion

In summary, we developed a data-driven framework called MetOncoFit to identify the common features of metabolic genes that are frequently dysregulated in tumors. Analysis of cancer-specific and pan cancer datasets revealed that tumor fitness is maximized by increasing expression of metabolic enzymes with high catalytic activity that are close to specific media components. These alterations result in increased flux through several metabolic pathways contributing to biomass synthesis, including glycolysis and the folate pathway. We also predicted that downregulation of metabolic enzymes in arginine and proline metabolism was correlated with increased patient survival; these pathways impact several redox and anaplerotic pathways. Overall, our analysis revealed new insights on the role of enzyme catalytic activity and the location of the enzyme in the metabolic network on tumor evolution and fitness.

## Methods

### Constructing cancer-specific MetOncoFit models

We constructed our MetOncoFit models using enzyme kinetics data from the SABIO enzyme biochemical database [22], metabolic network topology from the human metabolic network reconstruction RECON1 [16], copy number and mutation data from the COSMIC database of genetic alterations in cancers [24], transcriptomic database of NCI-60 cancer panel [23], and the multi-cancer patient survival database, PrognoScan [25]. Cancer-specific gene expression data for nine different cancer tissues (breast, central nervous system, colon, leukemia, melanoma, non-small cell lung, ovarian, prostate, and renal cancer) was taken from the NCBI Gene Expression Omnibus (GSE32474).

The number of unique metabolic genes varies with each cancer tissue model based on the available gene expression, survival, and CNV data. Genes with missing values were removed from the analysis. We did not fill in missing genes with a “NEUTRAL” label for two reasons. First, this would increase the class imbalance already present in the current models, inflating MetOncoFit’s prediction accuracies. Second, just because a gene was not measured in these datasets does not mean it has a neutral contribution towards the cancer tissue’s fitness. Our glioma model has the least number of unique metabolic genes (*n* = 190), while our pan cancer model has the maximum number of metabolic genes (*n* = 904) (Additional file 1: Table S2).

### Flux balance analysis of cancer cell line metabolic models

We focused on cancers exemplified in the NCI-60 cancer cell panel, which has been extensively studied using transcriptomic, proteomics, and metabolomics, and curated computer models of metabolism for these cell lines are available. The NCI-60 cancer cell line panel was representative of nine cancer types: breast cancer, B cell lymphoma, ovarian cancer, glioma, melanoma, prostate cancer, colon cancer, non-small cell lung cancer, and renal cell carcinoma. The cancer cell line specific cancer models were obtained from Yizhak et al.’s study; the models were built using the PRIME approach, which was shown to accurately recapitulate the metabolic state of various tumor cell lines by integrating cancer-specific transcriptomic and metabolic data [21]. The flux values were determined in each cancer-specific MetOncoFit model using flux balance analysis (FBA) from the Cobra Toolbox package (available in MATLAB and Python). To obtain a single unique flux solution for each cancer model, the sum total of fluxes through the metabolic network was minimized [50]. FBA was performed for each model to obtain the wild-type flux values for all reactions. Next, we performed single gene deletion analysis in each of the NCI-60 cancer cell line models and calculated the difference between wild-type and the cancer models. The average flux redistribution for each metabolic subsystem was calculated by taking the mean of all reaction flux differences corresponding to a given subsystem.

### Curating enzyme catalytic activity (*k*_cat_) data

The wild-type metabolic enzyme *k*_cat_ values were taken from the manually curated SABIO Biochemical Reaction database [22] using the UniProt identifiers associated with each human gene. Because the distribution of catalytic efficiency values spans a large range of values, we used the log_2_(*k*_cat_) value to train the model. For metabolic enzymes with multiple *k*_cat_ entries corresponding to different substrates, we used the median log_2_(*k*_cat_) value. If the kinetic data was not available for that particular metabolic enzyme, the value was set to the median log_2_(*k*_cat_) value across all metabolic enzymes in the dataset.

### Topological epicenter calculation

The topological distances of each gene to biomass components and media components were calculated using an unweighted directed graph of all reactant to product conversions. We used the Python library networkx to transform the metabolic network into a directed graph and the shortest_path function to obtain the shortest possible path between the source and the target. If a reaction is irreversible, all the reactants and products are connected by an edge from each reactant to product combination. If the reaction is reversible, the reactants and products are connected by two edges with both forward and backward directions. For each reaction in the graph, we computed the shortest path from the reaction to both medium components and biomass components. We then used the gene-reaction mapping relationship to find the distance between a gene and biomass or media components. The shortest path was used for genes that participate in more than one reaction. The sum total of such shortest distances to all biomass components, all media components, and sum of all topological distances were calculated to obtain the epicenter scores.

### Survival, CNV, and pan cancer class assignments and calculations

Cancer patient gene expression data was obtained from the PrognoScan database [25]. To designate the impact of a gene to the survival or mortality of a patient, three thresholds were set. For each gene in a tumor-specific context, the hazard ratio (HR) and the *p* value obtained from the Cox proportional hazard test were calculated across all cancers. If the HR was greater than or equal to 1.33 and the *p* value = 0.05 or less, the gene was designated as “UPREG.” If the HR was less than or equal to 0.75 and the *p* value = 0.05 or less, the gene was designated “DOWNREG.” The “NEUTRAL” class was designated for a gene if the HR between 0.75 and 1.33, or if the gene did not reach statistical significance. Some genes were detected multiple times in the same cohort. To reconcile potential class differences (i.e., a gene that had three entries corresponding to UPREG, UPREG, and NEUTRAL), we took the class that was observed the most frequently. In the previous example, we would label the gene UPREG. In the event of a tie or conflicting labels, we labeled the gene NEUTRAL.

We varied the HR thresholds for labeling between 0.5–2.0 and 0.90–1.10 as well to test different thresholds. We found that the model accuracy after 10-fold cross validation was lower in the scheme using a HR of 0.90–1.10, suggesting that the cutoffs are not sufficient to discriminate between labels. The more stringent cutoff of 0.5–2.0 has a clear clinical interpretation: a HR of 2.0 indicates that a gene is associated with twice the chance of dying compared to the control and vice versa. However, these labeling schemes resulted in less “DOWNREG” classifications across all cancer models. To balance predictive accuracy while controlling for class imbalance, we chose our final thresholds to be 0.75–1.33 (Additional file 1: Table S9).

Copy number variation (CNV) data from healthy and cancer patients were obtained from the Catalogue of Somatic Mutations in Cancer (COSMIC) database [24]. To determine if there was a gain or loss of CNV, we used COSMIC v83 definitions described below:

- To be classified as “GAIN” in copy number:
  - Average genome ploidy ≤ 2.7 AND total copy number ≥ 5, OR
  - Average genome ploidy > 2.7 AND total copy number ≥ 9
- To be classified “LOSS” in copy number:
  - Average genome ploidy ≤ 2.7 AND total copy number = 0, OR
  - Average genome ploidy > 2.7 AND total copy number < (average genome ploidy, 2.7)

Our target label for each gene prediction is the ratio of CNV GAIN/LOSS. We assigned our targets as follows: “NEUTRAL” if the CNV ratio was between 0.5 and 2.0 or if the total number of CNV measurements for the gene in a given cancer is less than 5. Otherwise, if the ratio was above 2.0, it was assigned “GAIN,” and less than 0.5 it was assigned “LOSS.”

To classify gene expression upregulation or downregulation for differential expression, we used the TCGA gene expression data from cancer patients for the 9 tumor models, available in the NCI-GDC Data Portal (https://portal.gdc.cancer.gov/). If a metabolic gene was identified multiple times in the same tissue, we took the median value as the final value. We took the log_2_ fold change of the tumor values over the normal gene expression values in healthy patients. A value of 2 or above was assigned as “UPREG,” while a value of − 2 or below was assigned as “DOWNREG.” A value between 2 and − 2 was assigned “NEUTRAL.”

### Data processing, analysis, and visualization

The analysis was performed using Python 3.6+ and several scientific computing libraries, notably scikit-learn. Since there is considerable variation in gene expression and other measures between cell lines, we use training data from all cell lines within a cancer type when developing a model for that cancer. For instance, for training the breast cancer model, we use five sets of data per gene from all five breast cancer cell lines (BT-549, HS-578-T, MCF7, MDA-MB-231, and T47D). The biological variation in the features between cell lines in a cancer type would minimize overfitting to any one cell line and will help in generalizing the model to novel data from other cell lines or by extension, patient-derived data. Features containing string data as the value such as the RECON1 subsystem feature were encoded with numerical values and were mapped back after making model predictions to get the true label association.

The resulting numeric array consisted of *n* genes in the cancer model × 142 features. These values were scaled by the interquartile range for each cancer modeling using the scikit-learn RobustScaler() function. To account for imbalanced classes within our dataset, we performed random oversampling to adjust the class distribution using the imbalanced-learn package. Finally, to generate the points in the dot plot, data from each gene-cell line pair was combined using a majority vote based on classification label or the medium value in the event of a tie. The figures in the manuscript were generated using the Matplotlib and Seaborn packages.

### MetOncoFit random forest classifier

We used copy number variation, TCGA differential expression, and PrognoScan patient survival as classification targets for MetOncoFit training. As shown in Fig. 1, the features described above are used with a random forest classifier from the scikit-learn Python package to predict each of the cancer fitness markers per gene. The overfitting to the training data was checked by testing against 30% of the initial data kept separate as validation data. The performance of the best parameters for each classification target via differential expression, CNV ratio, and over and under gene expression for survival benefit is given in Additional file 1: Tables S3–S5. Using random forests, we were able to measure and rank feature importance from classification using the Gini impurity index.

### Model validation

For each cancer model and target prediction, we calculated a confusion matrix using the test dataset and the model’s prediction. This comparison provides four metrics: true positives (TP), true negatives (TN), false positives (FP), and false negatives (FN) for each class. These values are used to calculate the precision, recall (sensitivity), specificity, and the area under the receiver operating characteristic curve (AUROC) score for each cancer model (Additional file 1: Table S3).

We found that MetOncoFit had high average precision and recall across all classes for predicting the cancer targets. We also calculated the harmonic average of the precision and recall (F1 score) and Matthew’s correlation coefficient (MCC), two measures that use the precision and recall values to evaluate the error in the model (Additional file 1: Tables S3–S5). We further calculated the upper-tailed *t* test *p* value and *Z*-score for the average accuracy in the test set based on a distribution of accuracy values obtained after 1000 iterations of shuffling the classification labels randomly. The *p* value for average accuracy across all models was less than 1× 10^−50^ (Additional file 1: Tables S3–S5).

To further test our model, we performed a holdout validation and 10-fold cross-validation and found that the sensitivity and specificity of prediction of each of the targets in eight different cancers is greater than 90% in both cross-validation and holdout validation. Taken together, the results suggest that the parameters used for classification are generalizable and show high performance in classifying the genes in each cancer model. Since the prediction targets and features are independent of each other, the high accuracy of the models indicates that biologically relevant features are being used for the classification.

To identify biases that are present within a given cancer cell line for the tissue models, we performed leave-one-cell line out analysis by calculating the model prediction accuracies after systematically removing a single cell line from the dataset (Additional file 1: Table S7). MetOncoFit had an average accuracy of 80% predicting all cancer markers across all cancers. The worst performing model was the prostate cancer model predicting CNV, which is due to the small sample size (*n* = 2 cell lines; DU-145 and PC-3). The best performing model was the renal cancer model for predicting patient survival (*n* = 7; 99% across all holdouts). Overall, we determined that the dataset we used to train MetOncoFit on is reasonable to make predictions for TCGA differential expression, CNV, and patient survival.

### Correlation between metabolic features and prognostic cancer targets

Pearson correlation coefficients were calculated between each metabolic/biochemical feature and its corresponding target—differential expression, copy number variation, and cancer patient survival. This determined if there was a positive or negative relationship between the feature and the predicted target. The upper and lower *R* values (*R* > 0.6 or *R* < − 0.6) were chosen as the cutoff for the positive (+) or negative (−) correlation.

## Supplementary information

**Additional file 1: Table S1.** Features. **Table S2.** Summary. **Table S3**. Differential expression prediction. **Table S4**. Copy number variation prediction. **Table S5.** Survival prediction. **Table S6.** Leave-one-feature-out. **Table S7.** Leave-one-cell-line-out. **Table S8.** Area under the receiver operating characteristic curve. **Table S9.** Hazard ratio check. **Table S10.** Breast Cancer. **Table S11.** Melanoma. **Table S12.** Lung Cancer. **Table S13.** B-cell lymphoma. **Table S14.** Ovarian Cancer. **Table S15.** Colorectal Cancer. **Table S16.** Renal Cancer. **Table S17.** Glioma. **Table S18.** Prostate Cancer.

**Additional file 2: Figure S1.** MetOncoFit predictions for gliomas. **Figure S2.** MetOncoFit predictions for colorectal cancer. **Figure S3.** MetOncoFit predictions for B-cell Lymphoma. **Figure S4.** MetOncoFit predictions for ovarian cancer. **Figure S5.** MetOncoFit predictions for prostate cancer. **Figure S6.** MetOncoFit predictions for renal cancer. **Figure S7.** MetOncoFit correctly predicts upregulation of RRM2. **Figure S8.** Gain in copy number for metabolic genes in the urea cycle is a recurring metabolic rewiring strategy in NSCLC. **Figure S9.** Incorporating TCGA gene expression fold change into the cancer models improves MetOncoFit’s predictive performance for copy number variation. **Figure S10.** Integrating TCGA fold change expression and copy number gain/loss ratios improve MetOncoFit predictions for cancer patient survival.

## Data Availability

The Python code, documentation, and relevant datasets for the MetOncoFit approach are available on GitHub (https://github.com/sriram-lab/MetOncoFit.git) and the interactive website (https://metoncofit.med.umich.edu). The compiled dataset used in this study is available at the Zenodo data repository (DOI 10.5281/zenodo.3520696), which includes the nine cancer tissue models and the pan cancer model. The input data for MetOncoFit for each cancer with all 142 features for each gene is also provided in the supplement (Additional file 1: Tables S10–S18).

## Abbreviations

AUROC curve: Area under the receiving operator characteristic curve
CNV: Copy number variation
CV: Cross validation
FBA: Flux balance analysis
FN: False negatives
FP: False positives
HR: Hazard ratio
MCC: Matthew’s correlation coefficient
TN: True negatives
TP: True positives
