# Supplementary material for: Common biochemical properties of metabolic genes recurrently dysregulated in tumors
Source: Cancer Metab. 2020 May 8;8:5. doi: 10.1186/s40170-020-0211-1 (PMC7206696; doi:10.1186/s40170-020-0211-1)
Supplement: Supplementary file 2 — Additional file 2: Figure S1. MetOncoFit predictions for gliomas. Figure S2. MetOncoFit predictions for colorectal cancer. Figure S3. MetOncoFit predictions for B-cell Lymphoma. Figure S4. MetOncoFit predictions for ovarian cancer. Figure S5. MetOncoFit predictions for prostate cancer. Figure S6. MetOncoFit predictions for renal cancer. Figure S7. MetOncoFit correctly predicts upregulation of RRM2. Figure S8. Gain in copy number for metabolic genes in the urea cycle is a recurring metabolic rewiring strategy in NSCLC. Figure S9. Incorporating TCGA gene expression fold change into the cancer models improves MetOncoFit’s predictive performance for copy number variation. Figure S10. Integrating TCGA fold change expression and copy number gain/loss ratios improve MetOncoFit predictions for cancer patient survival. [file 40170_2020_211_MOESM2_ESM.pdf]

Supplementary Information

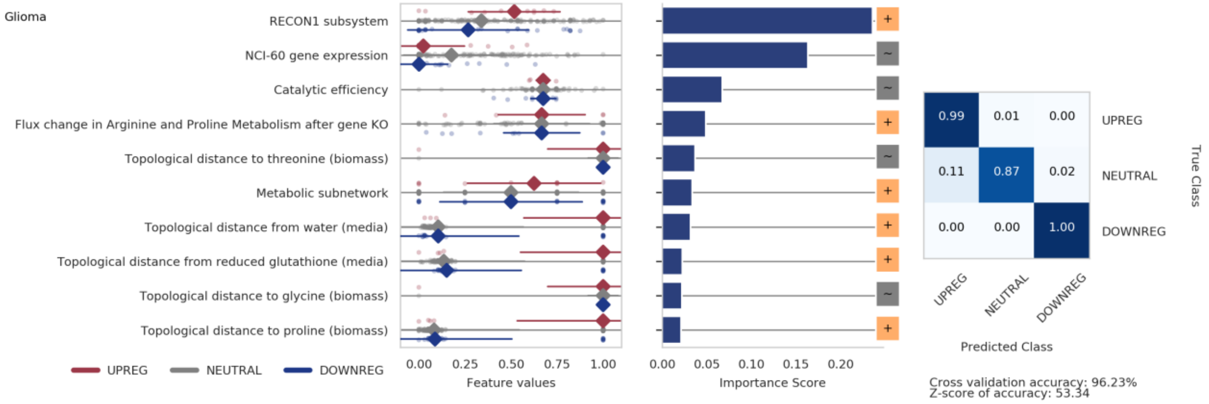

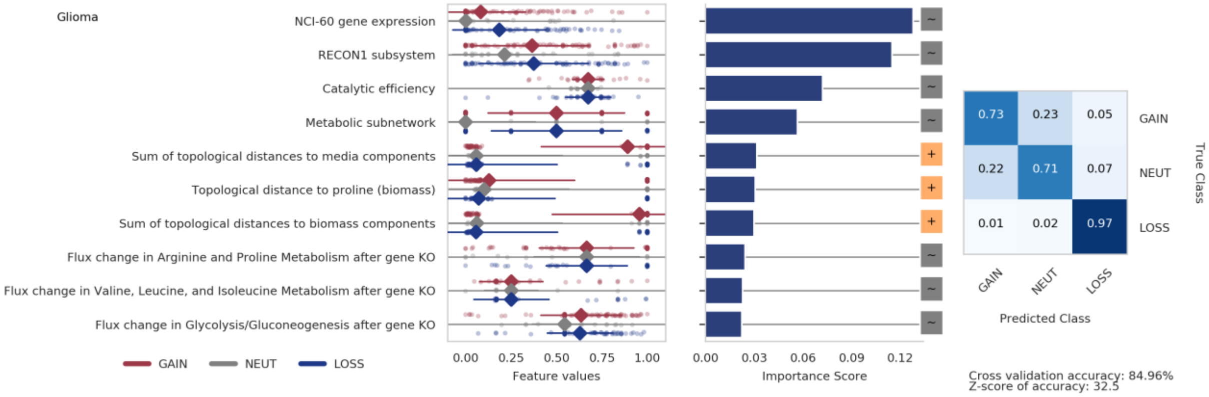

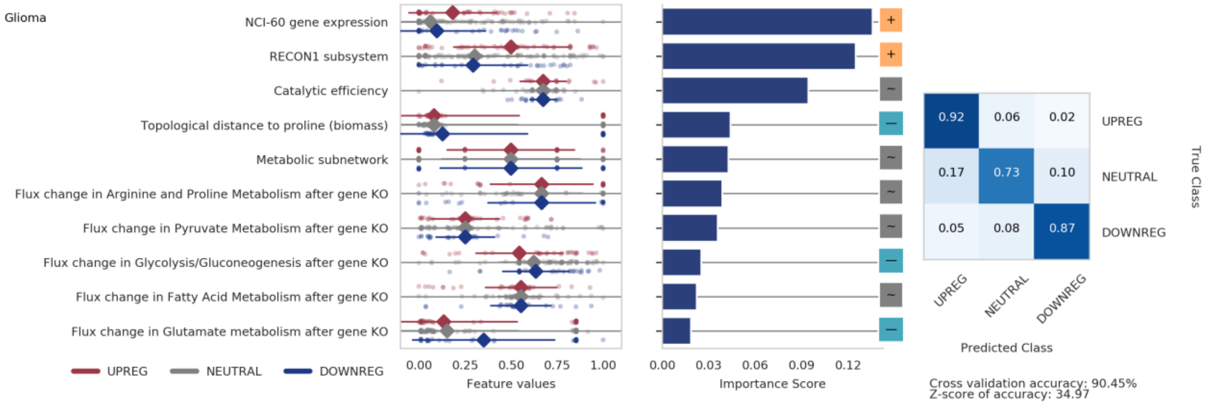

Supplementary Figure 1. MetOncoFit predictions for gliomas

Top | Differential expression: Several topological and metabolic flux features are predictive of dysregulated gene expression. Genes with high topological distances to biomass and medium components - proline and glutathione, were upregulated and those that are closer to these metabolites are downregulated. The 10-fold cross validation accuracy is 96%.

Middle | Copy number variation: Topological distances to biomass and medium components such as the amino acid - proline, are associated with a gain in copy number. 10-fold cross validation accuracy is 85%.

Bottom | Patient survival: Upregulation of genes topologically closer to proline are associated with increased patient survival. Flux through glycolysis and arginine/proline metabolism are also predictive of patient survival. 10-fold cross validation accuracy is 90%.

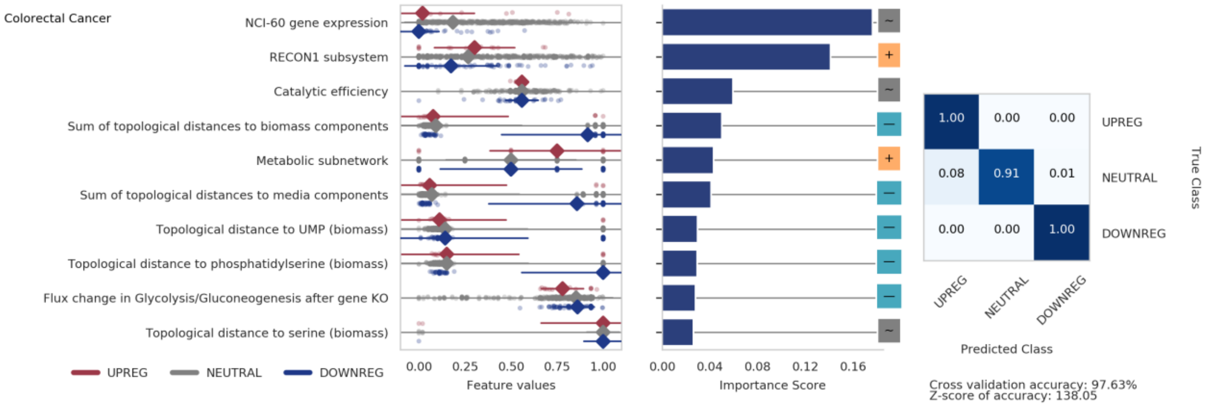
Colorectal Cancer
NCI-60 gene expression
RECON1 subsystem
Catalytic efficiency
Sum of topological distances to biomass components
Metabolic subnetwork
Sum of topological distances to media components
Topological distance to UMP (biomass)
Topological distance to phosphatidylserine (biomass)
Flux change in Glycolysis/Gluconeogenesis after gene KO
Topological distance to serine (biomass)
UPREG
NEUTRAL
DOWNREG
0.00 0.25 0.50 0.75 1.00
Feature values
0.00 0.04 0.08 0.12 0.16
Importance Score
1.00 0.00 0.00
0.08 0.91 0.01
0.00 0.00 1.00
UPREG
NEUTRAL
DOWNREG
True Class
Predicted Class
Cross validation accuracy: 97.63%
Z-score of accuracy: 138.05

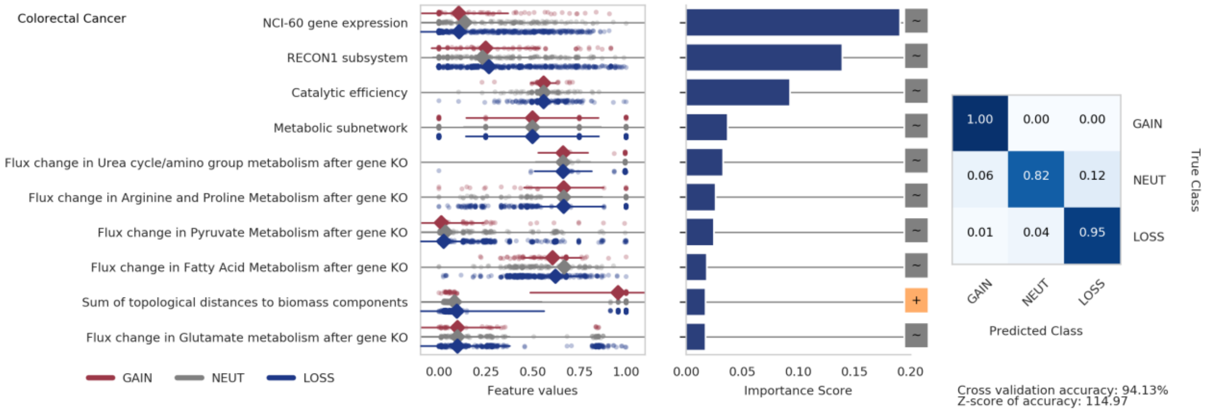
Colorectal Cancer
NCI-60 gene expression
RECON1 subsystem
Catalytic efficiency
Metabolic subnetwork
Flux change in Urea cycle/amino group metabolism after gene KO
Flux change in Arginine and Proline Metabolism after gene KO
Flux change in Pyruvate Metabolism after gene KO
Flux change in Fatty Acid Metabolism after gene KO
Sum of topological distances to biomass components
Flux change in Glutamate metabolism after gene KO
GAIN
NEUT
LOSS
0.00 0.25 0.50 0.75 1.00
Feature values
0.00 0.05 0.10 0.15 0.20
Importance Score
1.00 0.00 0.00
0.06 0.82 0.12
0.01 0.04 0.95
GAIN
NEUT
LOSS
True Class
Predicted Class
Cross validation accuracy: 94.13%
Z-score of accuracy: 114.97

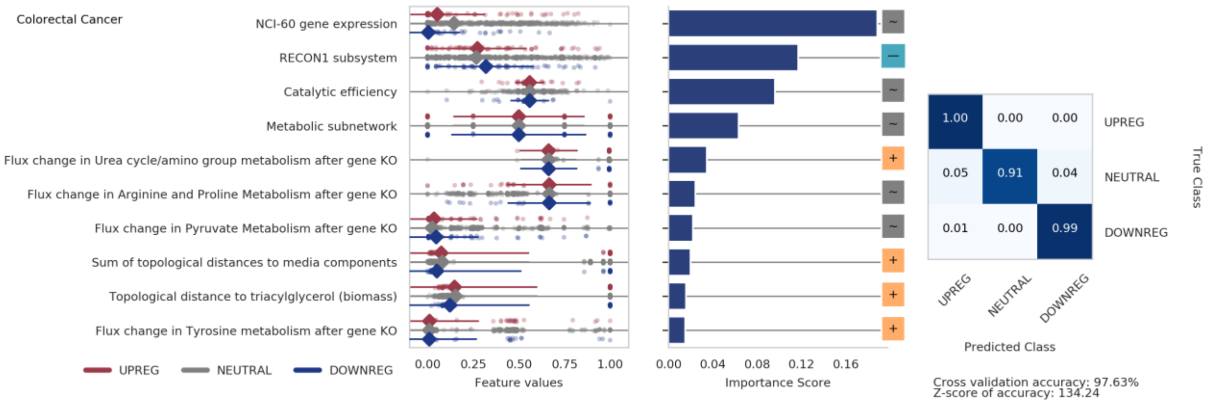
Colorectal Cancer
NCI-60 gene expression
RECON1 subsystem
Catalytic efficiency
Metabolic subnetwork
Flux change in Urea cycle/amino group metabolism after gene KO
Flux change in Arginine and Proline Metabolism after gene KO
Flux change in Pyruvate Metabolism after gene KO
Sum of topological distances to media components
Topological distance to triacylglycerol (biomass)
Flux change in Tyrosine metabolism after gene KO
UPREG
NEUTRAL
DOWNREG
0.00 0.25 0.50 0.75 1.00
Feature values
0.00 0.04 0.08 0.12 0.16
Importance Score
1.00 0.00 0.00
0.05 0.91 0.04
0.01 0.00 0.99
UPREG
NEUTRAL
DOWNREG
True Class
Predicted Class
Cross validation accuracy: 97.63%
Z-score of accuracy: 134.24

Supplemental Figure 2. MetOncoFit predictions for colorectal cancer

Top | Differential expression: Similar to other cancers, several topological features are associated with differential expression in colon cancer. Genes with low topological distances to biomass components – UMP and phosphatidyl serine, were upregulated. 10-fold cross validation accuracy is 98%.

Middle | Copy number variation: The biomass epicenter score is associated with a gain in copy number, genes that were farther from the network center showed a significant gain in copy number. Fatty acid metabolism showed a non-linear association with copy number, wherein genes that decreased flux through this pathway displayed copy number alterations, which can be either a loss or gain in copy number. 10-fold cross validation accuracy is 94%.

Bottom | Patient survival: Flux change in several metabolic pathways, including urea cycle, arginine and proline metabolism are predictive of patient survival. The sum of topological distances to biomass components are associated with negative patient outcomes; down regulation of genes closer to the network center was associated with positive patient survival. 10-fold cross validation accuracy is 98%.

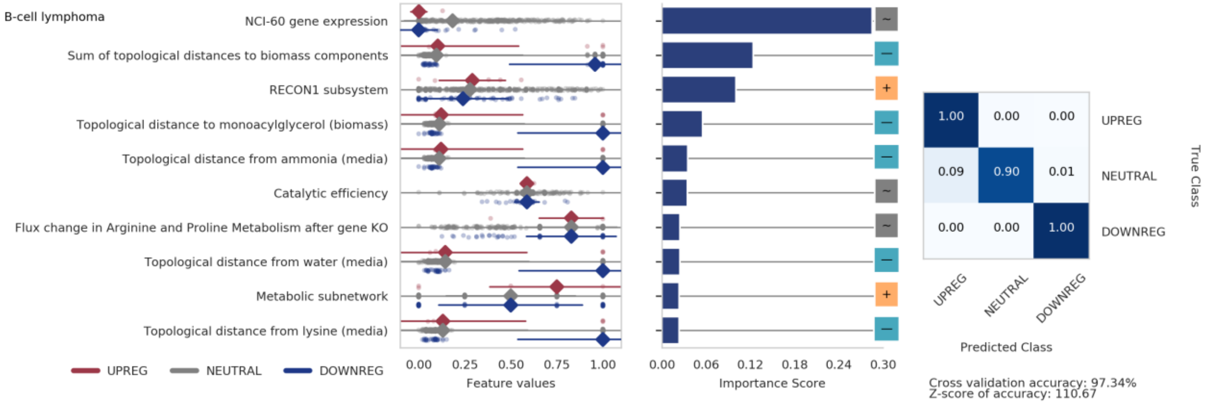
B-cell lymphoma
NCI-60 gene expression
Sum of topological distances to biomass components
RECON1 subsystem
Topological distance to monoacylglycerol (biomass)
Topological distance from ammonia (media)
Catalytic efficiency
Flux change in Arginine and Proline Metabolism after gene KO
Topological distance from water (media)
Metabolic subnetwork
Topological distance from lysine (media)
UPREG
NEUTRAL
DOWNREG
0.00 0.25 0.50 0.75 1.00
Feature values
0.00 0.06 0.12 0.18 0.24 0.30
Importance Score
1.00 0.00 0.00
0.09 0.90 0.01
0.00 0.00 1.00
UPREG
NEUTRAL
DOWNREG
True Class
Predicted Class
Cross validation accuracy: 97.34%
Z-score of accuracy: 110.67

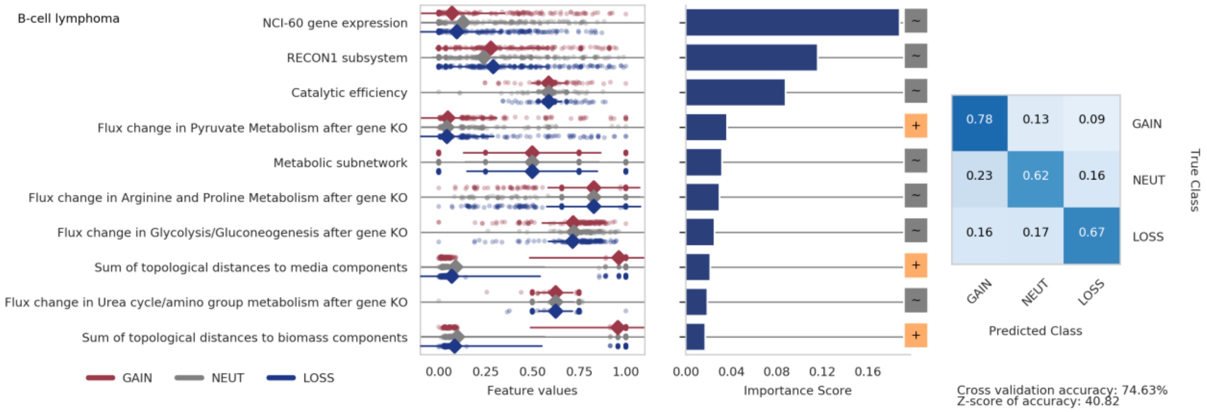
B-cell lymphoma
NCI-60 gene expression
RECON1 subsystem
Catalytic efficiency
Flux change in Pyruvate Metabolism after gene KO
Metabolic subnetwork
Flux change in Arginine and Proline Metabolism after gene KO
Flux change in Glycolysis/Gluconeogenesis after gene KO
Sum of topological distances to media components
Flux change in Urea cycle/amino group metabolism after gene KO
Sum of topological distances to biomass components
GAIN
NEUT
LOSS
0.00 0.25 0.50 0.75 1.00
Feature values
0.00 0.04 0.08 0.12 0.16
Importance Score
0.78 0.13 0.09
0.23 0.62 0.16
0.16 0.17 0.67
GAIN
NEUT
LOSS
True Class
Predicted Class
Cross validation accuracy: 74.63%
Z-score of accuracy: 40.82

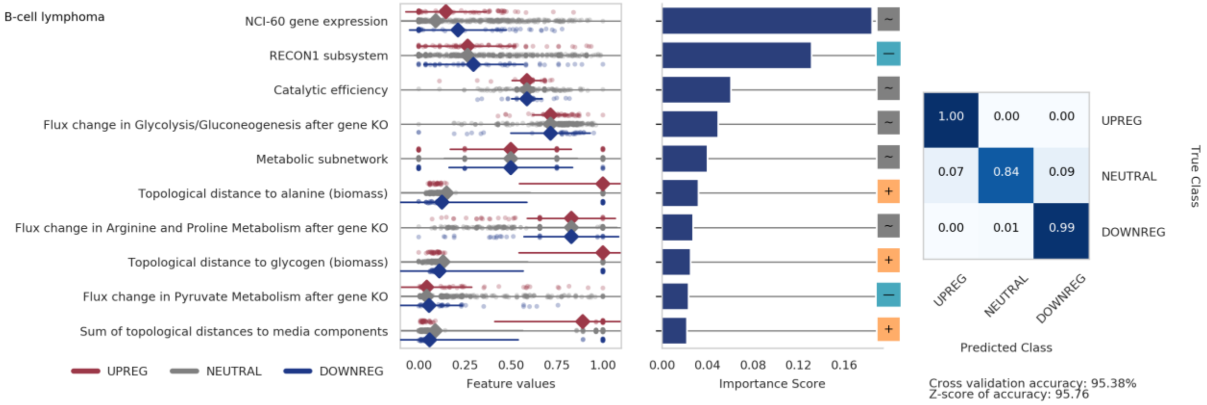
B-cell lymphoma
NCI-60 gene expression
RECON1 subsystem
Catalytic efficiency
Flux change in Glycolysis/Gluconeogenesis after gene KO
Metabolic subnetwork
Topological distance to alanine (biomass)
Flux change in Arginine and Proline Metabolism after gene KO
Topological distance to glycogen (biomass)
Flux change in Pyruvate Metabolism after gene KO
Sum of topological distances to media components
UPREG
NEUTRAL
DOWNREG
0.00 0.25 0.50 0.75 1.00
Feature values
0.00 0.04 0.08 0.12 0.16
Importance Score
1.00 0.00 0.00
0.07 0.84 0.09
0.00 0.01 0.99
UPREG
NEUTRAL
DOWNREG
True Class
Predicted Class
Cross validation accuracy: 95.38%
Z-score of accuracy: 95.76

Supplemental Figure 3. MetOncoFit predictions for B-cell Lymphoma

Top | Differential expression: Several topological features are associated with differential expression in B-cell Lymphoma cancer. Genes topologically closer to biomass and media components – lysine, ammonia and monoacyl glycerol, were upregulated.10-fold cross validation accuracy is 97%.

Middle | Copy number variation: Increased flux through pyruvate metabolism and high biomass and media epicenter scores are associated with a gain in copy number. 10-fold cross validation accuracy is 75%.

Bottom | Patient survival: Flux through pyruvate metabolism is associated with patient mortality; further downregulation of genes topologically closer to alanine and glycogen are associated with positive patient survival. 10-fold cross validation accuracy is 95%.

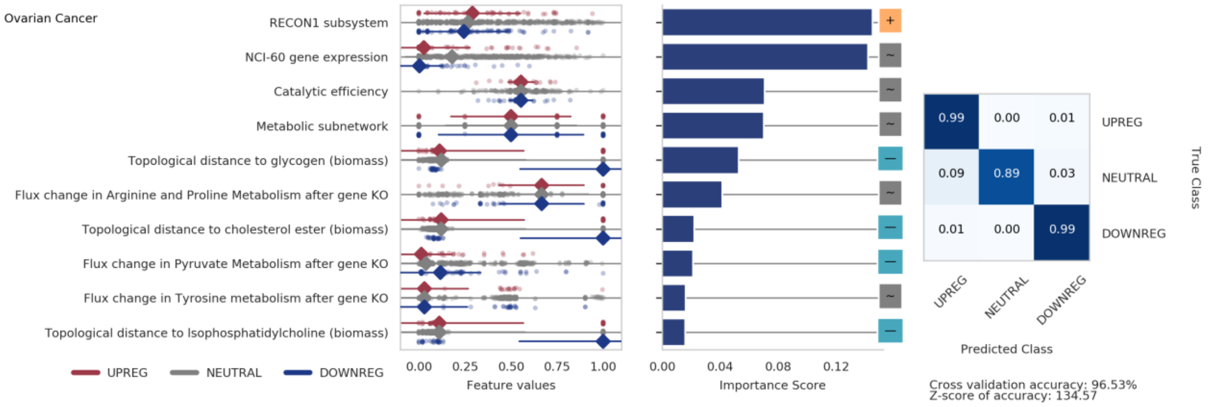
Ovarian Cancer
RECON1 subsystem
NCI-60 gene expression
Catalytic efficiency
Metabolic subnetwork
Topological distance to glycogen (biomass)
Flux change in Arginine and Proline Metabolism after gene KO
Topological distance to cholesterol ester (biomass)
Flux change in Pyruvate Metabolism after gene KO
Flux change in Tyrosine metabolism after gene KO
Topological distance to Isophosphatidylcholine (biomass)
UPREG
NEUTRAL
DOWNREG
Feature values
Importance Score
0.99 0.00 0.01
0.09 0.89 0.03
0.01 0.00 0.99
True Class
Predicted Class
Cross validation accuracy: 96.53%
Z-score of accuracy: 134.57

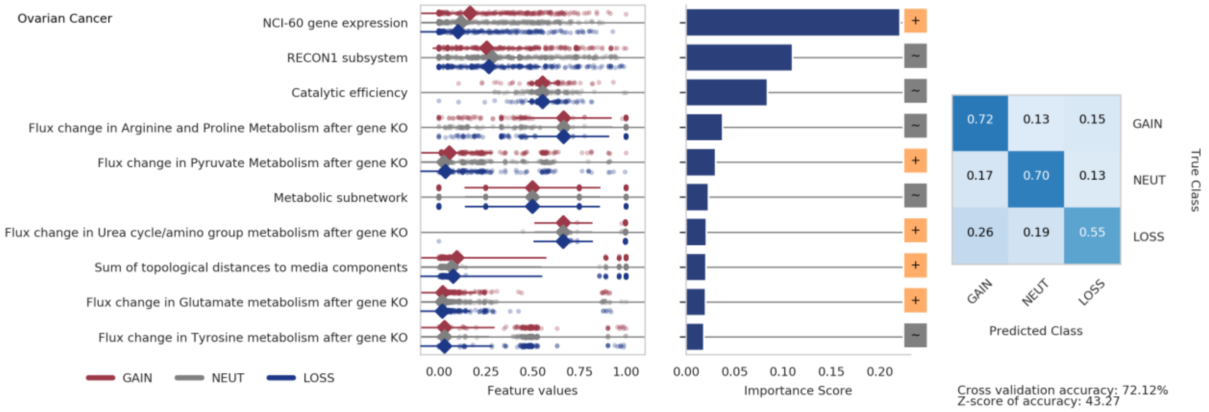
Ovarian Cancer
NCI-60 gene expression
RECON1 subsystem
Catalytic efficiency
Flux change in Arginine and Proline Metabolism after gene KO
Flux change in Pyruvate Metabolism after gene KO
Metabolic subnetwork
Flux change in Urea cycle/amino group metabolism after gene KO
Sum of topological distances to media components
Flux change in Glutamate metabolism after gene KO
Flux change in Tyrosine metabolism after gene KO
GAIN
NEUT
LOSS
Feature values
Importance Score
0.72 0.13 0.15
0.17 0.70 0.13
0.26 0.19 0.55
True Class
Predicted Class
Cross validation accuracy: 72.12%
Z-score of accuracy: 43.27

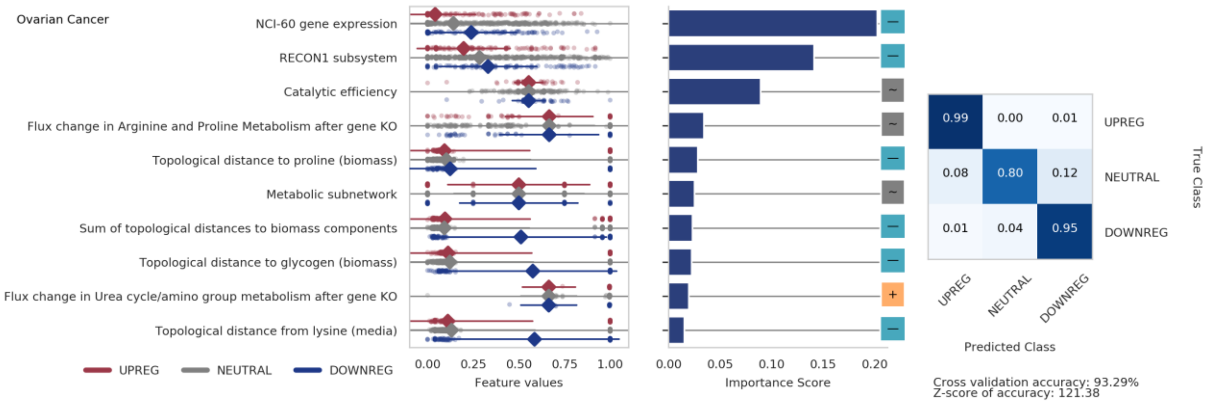
Ovarian Cancer
NCI-60 gene expression
RECON1 subsystem
Catalytic efficiency
Flux change in Arginine and Proline Metabolism after gene KO
Topological distance to proline (biomass)
Metabolic subnetwork
Sum of topological distances to biomass components
Topological distance to glycogen (biomass)
Flux change in Urea cycle/amino group metabolism after gene KO
Topological distance from lysine (media)
UPREG
NEUTRAL
DOWNREG
Feature values
Importance Score
0.99 0.00 0.01
0.08 0.80 0.12
0.01 0.04 0.95
True Class
Predicted Class
Cross validation accuracy: 93.29%
Z-score of accuracy: 121.38

Supplemental Figure 4. MetOncoFit predictions for ovarian cancer

Top | Differential expression: Similar to other cancers, several topological features are associated with differential expression in colon cancer. Genes with low topological distances to biomass components – cholesterol, glycogen and Isophosphatidyl choline, were upregulated. 10-fold cross validation accuracy is 97%.

Middle | Copy number variation: Genes with high media epicenter scores are associated with a gain in copy number. 10-fold cross validation accuracy is 72%.

Bottom | Patient survival: Metabolic flux through arginine and proline metabolism, and urea cycle are predictive of patient survival. 10-fold cross validation accuracy is 93%.

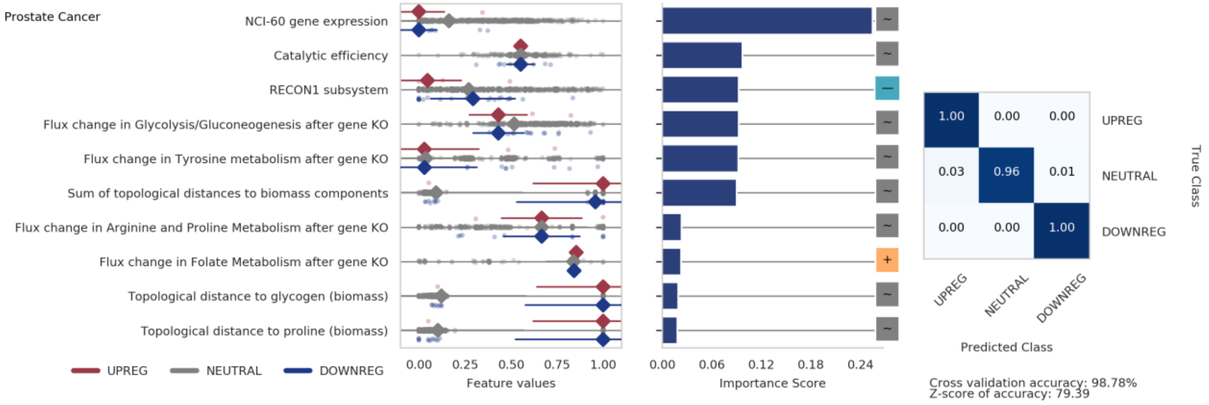
Prostate Cancer
NCI-60 gene expression
Catalytic efficiency
RECON1 subsystem
Flux change in Glycolysis/Gluconeogenesis after gene KO
Flux change in Tyrosine metabolism after gene KO
Sum of topological distances to biomass components
Flux change in Arginine and Proline Metabolism after gene KO
Flux change in Folate Metabolism after gene KO
Topological distance to glycogen (biomass)
Topological distance to proline (biomass)
UPREG NEUTRAL DOWNREG
0.00 0.25 0.50 0.75 1.00
Feature values
0.00 0.06 0.12 0.18 0.24
Importance Score
1.00 0.00 0.00 UPREG
0.03 0.96 0.01 NEUTRAL
0.00 0.00 1.00 DOWNREG
True Class
UPREG NEUTRAL DOWNREG
Predicted Class
Cross validation accuracy: 98.78%
Z-score of accuracy: 79.39

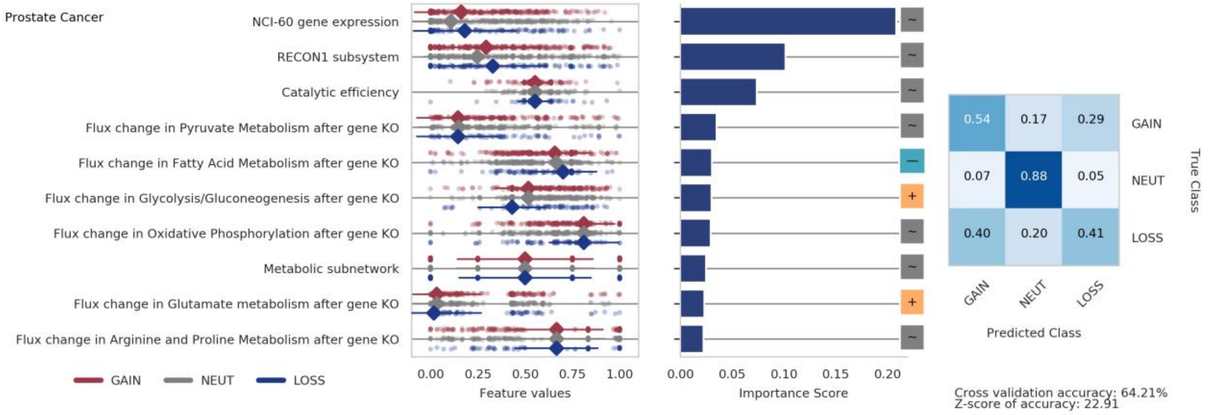
Prostate Cancer
NCI-60 gene expression
RECON1 subsystem
Catalytic efficiency
Flux change in Pyruvate Metabolism after gene KO
Flux change in Fatty Acid Metabolism after gene KO
Flux change in Glycolysis/Gluconeogenesis after gene KO
Flux change in Oxidative Phosphorylation after gene KO
Metabolic subnetwork
Flux change in Glutamate metabolism after gene KO
Flux change in Arginine and Proline Metabolism after gene KO
GAIN NEUT LOSS
0.00 0.25 0.50 0.75 1.00
Feature values
0.00 0.05 0.10 0.15 0.20
Importance Score
0.54 0.17 0.29 GAIN
0.07 0.88 0.05 NEUT
0.40 0.20 0.41 LOSS
True Class
GAIN NEUT LOSS
Predicted Class
Cross validation accuracy: 64.21%
Z-score of accuracy: 22.91

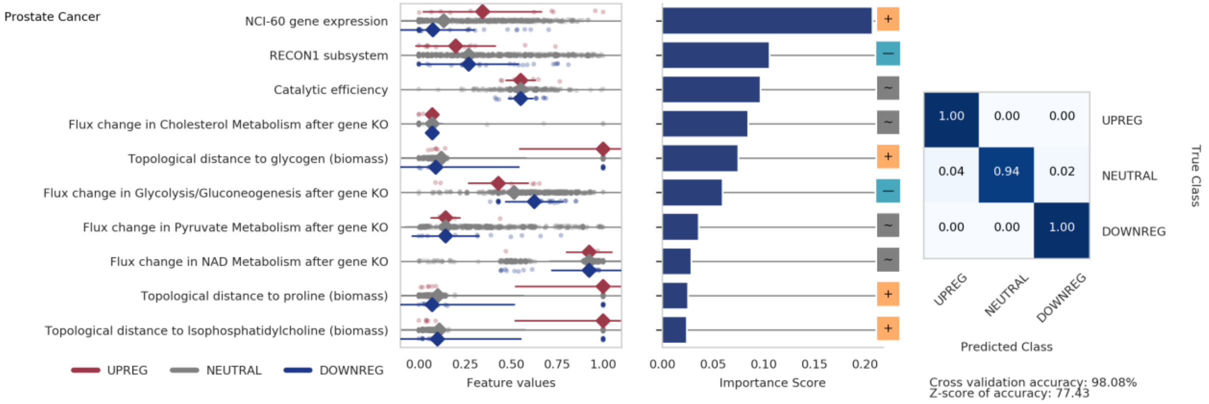
Prostate Cancer
NCI-60 gene expression
RECON1 subsystem
Catalytic efficiency
Flux change in Cholesterol Metabolism after gene KO
Topological distance to glycogen (biomass)
Flux change in Glycolysis/Gluconeogenesis after gene KO
Flux change in Pyruvate Metabolism after gene KO
Flux change in NAD Metabolism after gene KO
Topological distance to proline (biomass)
Topological distance to lsophosphatidylcholine (biomass)
UPREG NEUTRAL DOWNREG
0.00 0.25 0.50 0.75 1.00
Feature values
0.00 0.05 0.10 0.15 0.20
Importance Score
1.00 0.00 0.00 UPREG
0.04 0.94 0.02 NEUTRAL
0.00 0.00 1.00 DOWNREG
True Class
UPREG NEUTRAL DOWNREG
Predicted Class
Cross validation accuracy: 98.08%
Z-score of accuracy: 77.43

Supplemental Figure 5. MetOncoFit predictions for prostate cancer

Top | Differential expression: Flux through folate metabolism is associated with gene upregulation. Glycolytic flux showed a non-linear association with differential gene expression, wherein genes that decreased flux through this pathway were either up- and down-regulated. Hence flux through this pathway is predictive of dysregulation but not in a direct linear fashion. 10-fold cross validation accuracy is 99%.

Middle | Copy number variation: Increased flux through glutamate metabolism and glycolysis is associated with a gain in copy number. Metabolic flux through fatty acid metabolism is associated with copy number loss. 10-fold cross validation accuracy is 64%.

Bottom | Patient survival: Several topological features are associated with both positive patient outcomes and patient mortality. Downregulation of genes with low topological distances to biomass components – proline, glycogen and Isophosphatidyl choline, is associated with better patient survival. 10-fold cross validation accuracy is 98%.

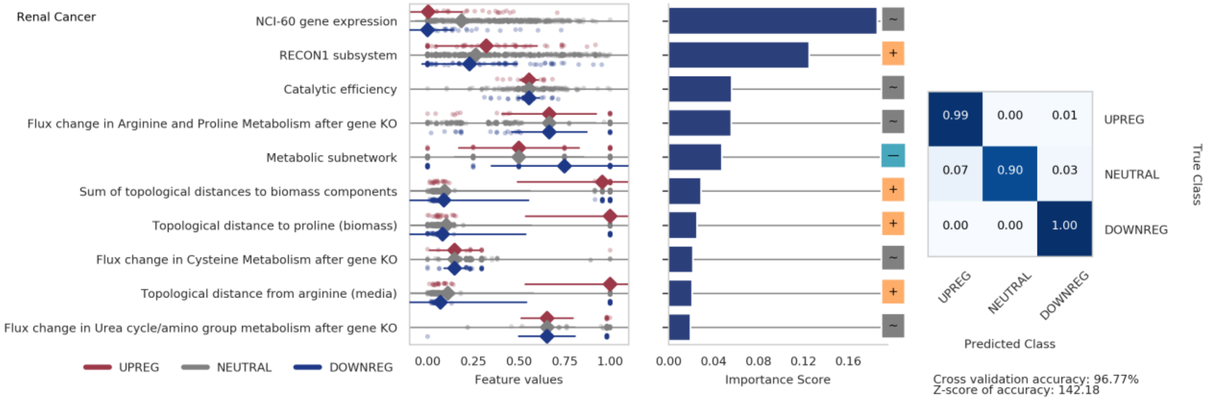
Renal Cancer
NCI-60 gene expression
RECON1 subsystem
Catalytic efficiency
Flux change in Arginine and Proline Metabolism after gene KO
Metabolic subnetwork
Sum of topological distances to biomass components
Topological distance to proline (biomass)
Flux change in Cysteine Metabolism after gene KO
Topological distance from arginine (media)
Flux change in Urea cycle/amino group metabolism after gene KO
UPREG NEUTRAL DOWNREG
0.00 0.25 0.50 0.75 1.00
Feature values
0.00 0.04 0.08 0.12 0.16
Importance Score
0.99 0.00 0.01 UPREG
0.07 0.90 0.03 NEUTRAL
0.00 0.00 1.00 DOWNREG
True Class
UPREG NEUTRAL DOWNREG
Predicted Class
Cross validation accuracy: 96.77%
Z-score of accuracy: 142.18

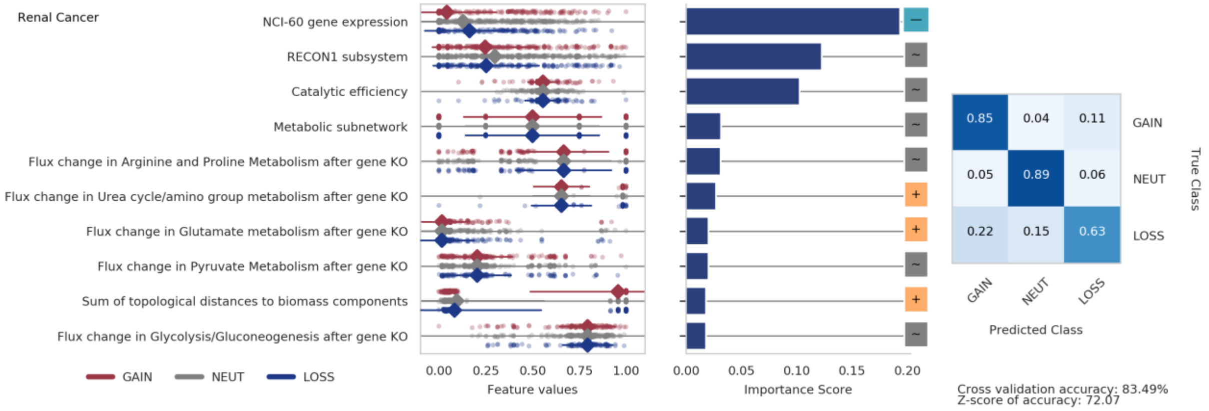
Renal Cancer
NCI-60 gene expression
RECON1 subsystem
Catalytic efficiency
Metabolic subnetwork
Flux change in Arginine and Proline Metabolism after gene KO
Flux change in Urea cycle/amino group metabolism after gene KO
Flux change in Glutamate metabolism after gene KO
Flux change in Pyruvate Metabolism after gene KO
Sum of topological distances to biomass components
Flux change in Glycolysis/Gluconeogenesis after gene KO
GAIN NEUT LOSS
0.00 0.25 0.50 0.75 1.00
Feature values
0.00 0.05 0.10 0.15 0.20
Importance Score
0.85 0.04 0.11 GAIN
0.05 0.89 0.06 NEUT
0.22 0.15 0.63 LOSS
True Class
GAIN NEUT LOSS
Predicted Class
Cross validation accuracy: 83.49%
Z-score of accuracy: 72.07

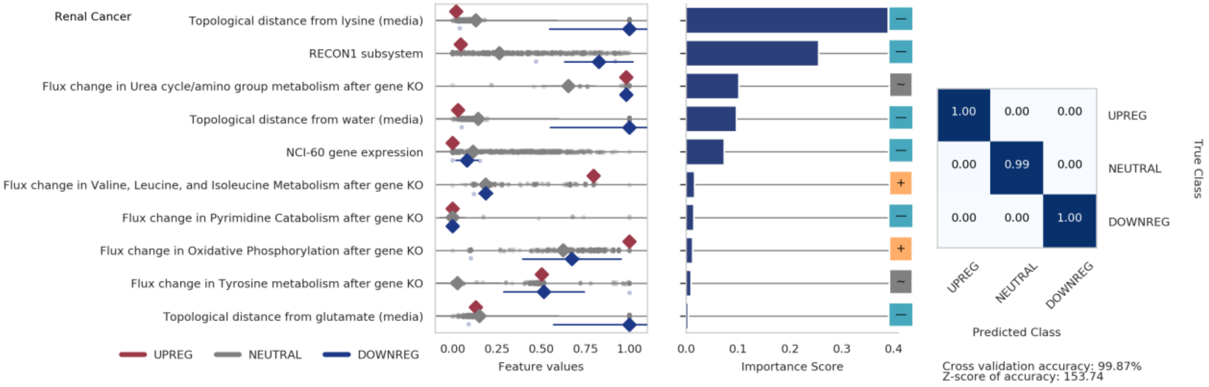
Renal Cancer
Topological distance from lysine (media)
RECON1 subsystem
Flux change in Urea cycle/amino group metabolism after gene KO
Topological distance from water (media)
NCI-60 gene expression
Flux change in Valine, Leucine, and Isoleucine Metabolism after gene KO
Flux change in Pyrimidine Catabolism after gene KO
Flux change in Oxidative Phosphorylation after gene KO
Flux change in Tyrosine metabolism after gene KO
Topological distance from glutamate (media)
UPREG NEUTRAL DOWNREG
0.00 0.25 0.50 0.75 1.00
Feature values
0.0 0.1 0.2 0.3 0.4
Importance Score
1.00 0.00 0.00 UPREG
0.00 0.99 0.00 NEUTRAL
0.00 0.00 1.00 DOWNREG
True Class
UPREG NEUTRAL DOWNREG
Predicted Class
Cross validation accuracy: 99.87%
Z-score of accuracy: 153.74

Supplemental Figure 6. MetOncoFit predictions for renal cancer

Top | Differential expression: Several topological and metabolic flux features are associated with gene upregulation. For example, genes away from the network center (i.e. high topological distance to all biomass components) were likely to be upregulated, while those closer to the amino-acid - proline are downregulated. 10-fold cross validation accuracy is 97%.

Middle | Copy number variation: Flux through arginine and proline metabolism, urea cycle and glutamate pathway are predictive of copy number alterations. Similar to other cancers, genes away from the network center were likely to show a gain in copy number. 10-fold cross validation accuracy is 83%.

Bottom | Patient survival: Upregulation of genes with low topological distances to biomass components – lysine, water and glutamate, is associated with better patient survival. Increased flux through oxidative phosphorylation pathway is also predictive of increased patient survival. 10-fold cross validation accuracy is 99%.

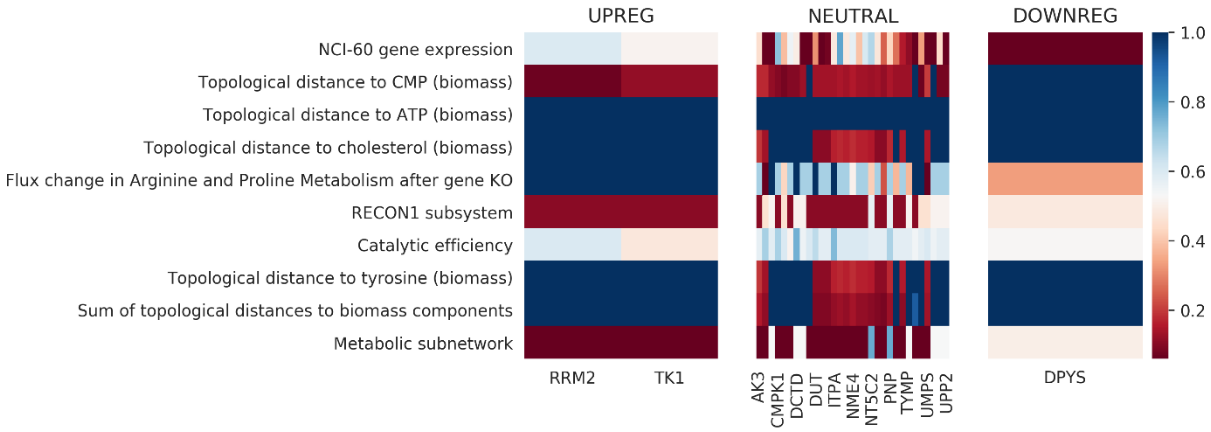
UPREG
NEUTRAL
DOWNREG
NCI-60 gene expression
Topological distance to CMP (biomass)
Topological distance to ATP (biomass)
Topological distance to cholesterol (biomass)
Flux change in Arginine and Proline Metabolism after gene KO
RECON1 subsystem
Catalytic efficiency
Topological distance to tyrosine (biomass)
Sum of topological distances to biomass components
Metabolic subnetwork
RRM2
TK1
AK3
CMPK1
DCTD
DUT
ITPA
NME4
NT5C2
PNP
TYMP
UMPS
UPP2
DPYS
1.0
0.8
0.6
0.4
0.2

Supplemental Figure 7. MetOncoFit correctly predicts upregulation of RRM2
Genes selected in the heatmaps are from the KEGG gene set for Pyrimidine metabolism. MetOncoFit correctly predicted that RRM2 is dysregulated based on **a)** its short topological distance to CMP, **b)** upregulation in breast cancer cell lines, and **c)** high catalytic efficiency.

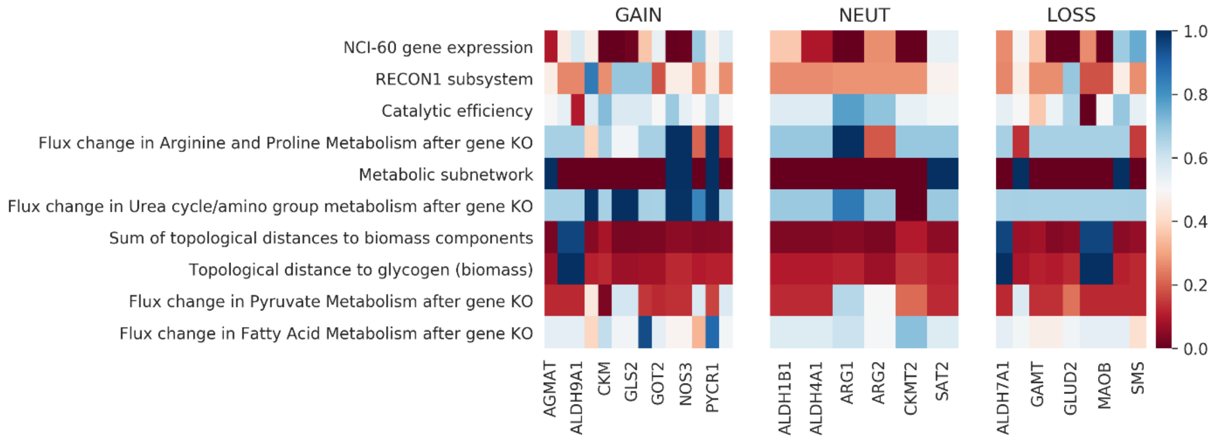
GAIN
NEUT
LOSS
NCI-60 gene expression
RECON1 subsystem
Catalytic efficiency
Flux change in Arginine and Proline Metabolism after gene KO
Metabolic subnetwork
Flux change in Urea cycle/amino group metabolism after gene KO
Sum of topological distances to biomass components
Topological distance to glycogen (biomass)
Flux change in Pyruvate Metabolism after gene KO
Flux change in Fatty Acid Metabolism after gene KO
AGMAT
ALDH9A1
CKM
GLS2
GOT2
NOS3
PYCR1
ALDH1B1
ALDH4A1
ARG1
ARG2
CKMT2
SAT2
ALDH7A1
GAMT
GLUD2
MAOB
SMS
1.0
0.8
0.6
0.4
0.2
0.0

Supplemental Figure 8. Gain in copy number for metabolic genes in the urea cycle is a recurring metabolic rewiring strategy in NSCLC

Genes selected in the heatmaps are from the KEGG gene set for arginine and proline metabolism, which contains the urea cycle. Several genes that directly participate in or are proximal to the urea cycle, including GLS2, GOT2, and NOS3 display a gain in copy number and have high metabolic flux through the urea cycle.

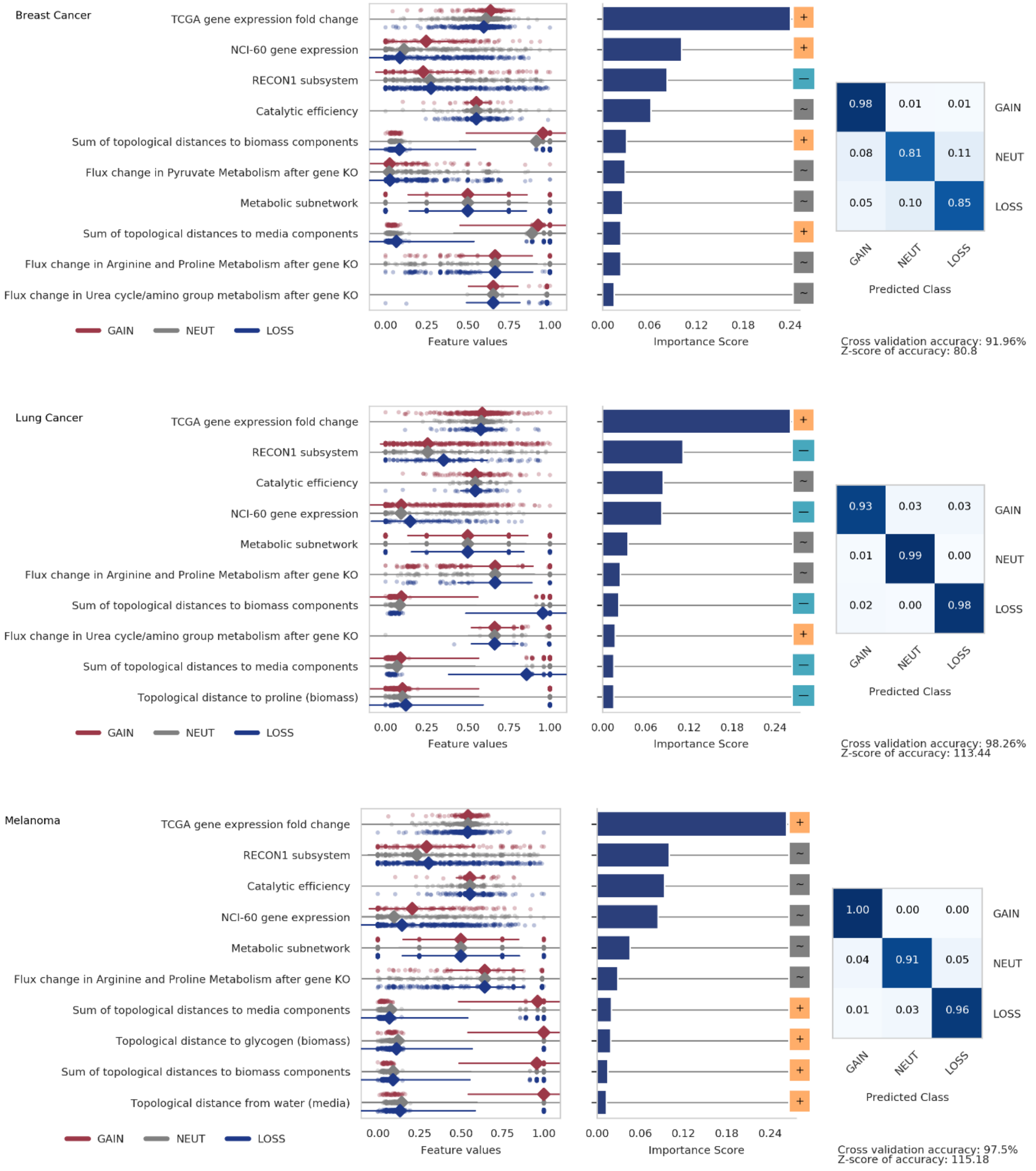

Breast Cancer
TCGA gene expression fold change
NCI-60 gene expression
RECON1 subsystem
Catalytic efficiency
Sum of topological distances to biomass components
Flux change in Pyruvate Metabolism after gene KO
Metabolic subnetwork
Sum of topological distances to media components
Flux change in Arginine and Proline Metabolism after gene KO
Flux change in Urea cycle/amino group metabolism after gene KO
GAIN
NEUT
LOSS
Feature values
Importance Score
0.98 0.01 0.01
0.08 0.81 0.11
0.05 0.10 0.85
True Class
Predicted Class
Cross validation accuracy: 91.96%
Z-score of accuracy: 80.8
Lung Cancer
TCGA gene expression fold change
RECON1 subsystem
Catalytic efficiency
NCI-60 gene expression
Metabolic subnetwork
Flux change in Arginine and Proline Metabolism after gene KO
Sum of topological distances to biomass components
Flux change in Urea cycle/amino group metabolism after gene KO
Sum of topological distances to media components
Topological distance to proline (biomass)
0.93 0.03 0.03
0.01 0.99 0.00
0.02 0.00 0.98
Cross validation accuracy: 98.26%
Z-score of accuracy: 113.44
Melanoma
TCGA gene expression fold change
RECON1 subsystem
Catalytic efficiency
NCI-60 gene expression
Metabolic subnetwork
Flux change in Arginine and Proline Metabolism after gene KO
Sum of topological distances to media components
Topological distance to glycogen (biomass)
Sum of topological distances to biomass components
Topological distance from water (media)
1.00 0.00 0.00
0.04 0.91 0.05
0.01 0.03 0.96
Cross validation accuracy: 97.5%
Z-score of accuracy: 115.18

Supplementary Figure 9. Incorporating TCGA gene expression fold change into the cancer models improves MetOncoFit's predictive performance for copy number variation.

High TCGA gene fold change was correlated with a gain in metabolic gene copy number for breast cancer, NSCLC, and melanoma. This analysis revealed several topological features including topological biomass epicenter scores, and metabolic flux through several pathways including arginine and proline metabolism as predictive of copy number variation in these cancers.

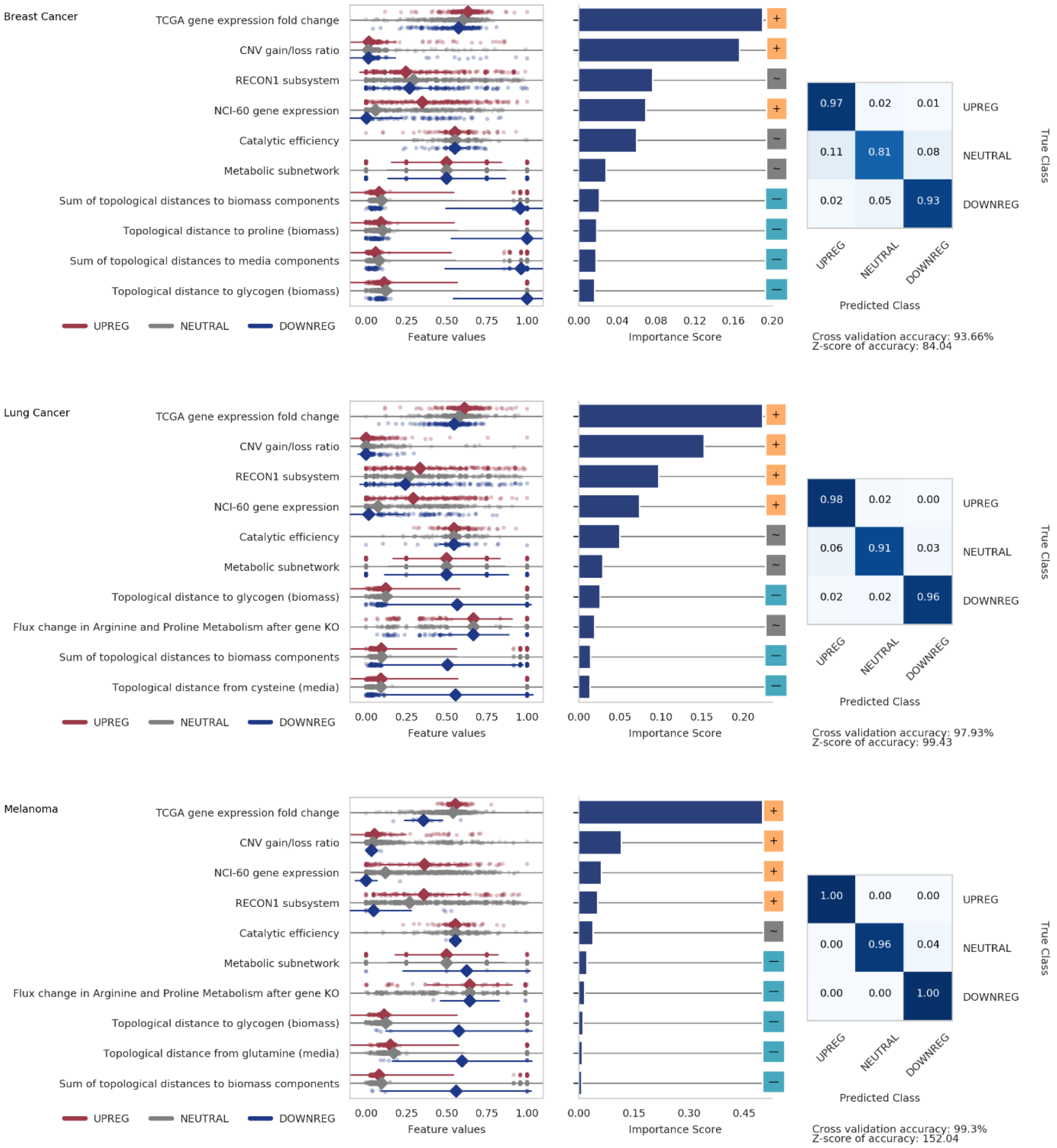
Breast Cancer
TCGA gene expression fold change
CNV gain/loss ratio
RECON1 subsystem
NCI-60 gene expression
Catalytic efficiency
Metabolic subnetwork
Sum of topological distances to biomass components
Topological distance to proline (biomass)
Sum of topological distances to media components
Topological distance to glycogen (biomass)
UPREG
NEUTRAL
DOWNREG
0.00 0.25 0.50 0.75 1.00
Feature values
0.00 0.04 0.08 0.12 0.16 0.20
Importance Score
0.97 0.02 0.01
0.11 0.81 0.08
0.02 0.05 0.93
True Class
Predicted Class
Cross validation accuracy: 93.66%
Z-score of accuracy: 84.04
Lung Cancer
TCGA gene expression fold change
CNV gain/loss ratio
RECON1 subsystem
NCI-60 gene expression
Catalytic efficiency
Metabolic subnetwork
Topological distance to glycogen (biomass)
Flux change in Arginine and Proline Metabolism after gene KO
Sum of topological distances to biomass components
Topological distance from cysteine (media)
0.00 0.05 0.10 0.15 0.20
0.98 0.02 0.00
0.06 0.91 0.03
0.02 0.02 0.96
Cross validation accuracy: 97.93%
Z-score of accuracy: 99.43
Melanoma
TCGA gene expression fold change
CNV gain/loss ratio
NCI-60 gene expression
RECON1 subsystem
Catalytic efficiency
Metabolic subnetwork
Flux change in Arginine and Proline Metabolism after gene KO
Topological distance to glycogen (biomass)
Topological distance from glutamine (media)
Sum of topological distances to biomass components
0.00 0.15 0.30 0.45
1.00 0.00 0.00
0.00 0.96 0.04
0.00 0.00 1.00
Cross validation accuracy: 99.3%
Z-score of accuracy: 152.04

Supplementary Figure 10. Integrating TCGA fold change expression and copy number gain/loss ratios improve MetOncoFit predictions for cancer patient survival

TCGA gene expression fold change and copy number values are the top predictors of cancer patient survival in breast cancer, NSCLC, and melanoma. NCI-60 gene expression, The topological biomass epicenter score, catalytic efficiency and flux through arginine and proline metabolism are also top predictors across all cancers.
